# Ongoing formation of felsic lower crustal channel by relamination in Zagros collision zone revealed from regional tomography

**DOI:** 10.1038/s41598-020-64946-w

**Published:** 2020-05-19

**Authors:** Amir Talebi, Ivan Koulakov, Ali Moradi, Habib Rahimi, Taras Gerya

**Affiliations:** 1 Institute of Geophysics. University of Tehran, Tehran, Iran; 2 Trofimuk Institute of Petroleum Geology and Geophysics, SB RAS, Prospekt Koptyuga, Novosibirsk, 630090 Russia; 3 Novosibirsk State University, Novosibirsk, Russia, Pirogova 2, Novosibirsk, 630090 Russia; 4 ETH Zurich, Department of Earth Sciences, Sonneggstrasse 5, Zurich, 8092 Switzerland

**Keywords:** Seismology, Tectonics

## Abstract

Complex interaction of rheologically contrasting layers within the lithosphere during the collision of continental plates leads to active faulting, which represents a serious hazard to the population and infrastructure. One of the collision scenarios presumes the existence of a middle-lower crustal channel composed of subducted silicic upper crustal rocks, which is thought to exist in the Tibetan-Himalayan system. Based on the results of seismic tomography, we argue that a similar mechanism of crustal channeling takes place beneath the Zagros mountain system in southwestern Iran. The 3D seismic velocity model reveals an inverted crustal architecture of the collision zone, in which the low-velocity felsic (granitic and sedimentary) upper crustal rocks of the Arabian plate form a seismically inactive lower crustal channel below the higher-velocity mafic (basaltic) middle-upper crustal layer of the Iranian crust. Based on existing numerical models, we suggest that the formation of the felsic channel is likely governed by separation (delamination) of the weak felsic upper crust of the subducting Arabian lithosphere and its ductile underplating under rheologically stronger upper-middle crust of the Iranian plate.

## Introduction

The collision of continental plates is a complex geodynamic process leading to strong tectonic activity that affects some densely populated areas in the world. The continental lithospheric plates consist of multiple compositionally contrasting crustal layers and the mantle lithosphere contains strongly variable rheological properties. Understanding the mechanisms of the interaction between the lithosphere layers during collision and shortening of continental plates is an on-going challenge that is actively discussed in the scientific literature^1^.

The existence of rheologically weak partially molten middle-lower crustal channels composed of subducted silicic upper crustal rocks has been proposed based on geological-geophysical data from modern and ancient continental collision zones such as the Tibetan-Himalayan system or Variscan orogeny^2–4^. However, the physical mechanism and the dynamics of formation of such channels remain enigmatic and have, so far, only been investigated on the basis of numerical modeling^2–4^. These models show that the formation of the felsic channel is likely governed by separation (delamination) of the weak felsic upper crust of the subducting plate and its ductile underplating under rheologically stronger upper-middle crust of the overriding plate. However, there are few areas in the world where such a mechanism can be directly observed in geophysical images^5,6^.

We propose that the mechanism of crustal channeling may take place beneath the Zagros mountain belt in the collision zone between the Arabian Plate and Central Iran. To investigate this hypothesis, we create a new 3D seismic velocity model of the crust and uppermost mantle beneath the southwestern part of Iran that reveal the details of the lithosphere structures in the colliding plates.

## Zagros: general information and previous studies

The Zagros orogenic belt was formed approximately 12 million years ago due to the convergence between the Arabian and Eurasian plates upon the closing of the Neo-Tethys Ocean. Although the precise time of the final phase of oceanic closure or initiation of collision is controversial^7–9^, the Zagros is categorized as one of the youngest such settings on Earth, at an early stage of collision^10^.

**Figure 1:**
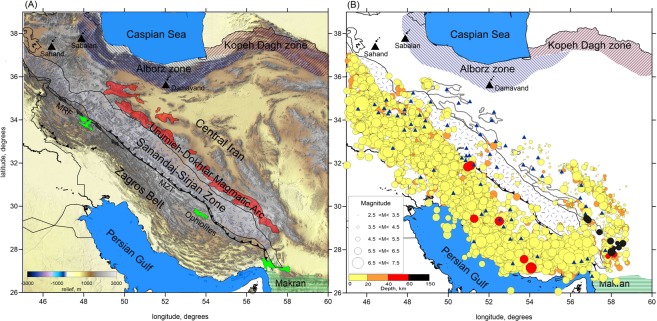
Structural features in the study area and data information. (**A**) The topography of Iranian plateau with main tectonic settings. Different structural elements related to the Zagros collision zone, from Southwest to Northeast, named the Zagros Fold and Thrust Belt (ZFTB); The Sanandaj–Sirjan Metamorphic Zone (SSZ); and the Urumieh–Dokhtar Magmatic Arc (UDMA). Black triangles indicate some inactive volcanoes in Iran. The main tectonic faults including the Main Zagros Thrust (MZT; or Main Zagros Reverse Fault) and the Main Recent Fault (MRF), are shown in the map. This map modified from the structural map of NGDIR (National Geoscience Database of Iran, http://www.ngdir.ir). (**B**) Green areas in the figure represent the ophiolites. (**B**) The distribution of events and stations used in this study from the merged Iranian Seismological Center, Institute of Geophysics, University of Tehran (IRSC, IGUT) and International Institute of Earthquake Engineering and Seismology (IIEES) of Iran catalogues. The events are plotted based on different sizes and depths according to the scales shown in the map. The figure was generated using the software Surfer (version 13, http://www.goldensoftware.com/products/surfer).

The Zagros extends for approximately 2000 km from eastern Turkey in the northwest, to the Makran subduction zone in southeastern Iran^11^. Geological observations show that the Zagros experienced complex deformation that formed several parallel tectonic structures^12^. From southwest to northeast (perpendicular to the main trend) the Zagros belt can be subdivided into three elongated zones: 1- the Zagros Fold and Thrust Belt (ZFTB); 2- the Sanandaj–Sirjan Metamorphic Zone (SSZ); and 3- the Urumieh–Dokhtar Magmatic Arc (UDMA) (Fig. 1A). In the northeast the ZFTB is bounded by an active thrust fault, the Main Zagros Thrust (MZT), which is believed to be the suture zone between the Arabian and Iranian plates^13–15^. The ZFTB is covered by 13–14 km of sediments of Permo–Triassic to Late Cretaceous/Paleocene ages that were folded in the NW–SE direction after the Mio–Pliocene^7,16^.

The SSZ is mainly composed of Precambrian metamorphic rocks. The presence of ophiolite sequences along the MZT shows that the SSZ experienced various metamorphic processes during the subduction of the Tethyan Ocean beneath the Iranian microplate^14,17^. The SSZ consists of sedimentary and Paleozoic to Cretaceous metamorphic rocks created in the previous active margin of an Iranian microplate which drifted during the Late Jurassic^7^. These metamorphics are overlain by unconformable Barremo–Aptian Orbitolina limestones typical of Central Iran sedimentation. During most of the second half of the Mesozoic, the SSZ represented an active Andean-like margin whose calc–alkaline magmatic activity progressively shifted northward^14^. It is today moving northward at 14 mm/year with respect to stable Eurasia as a fairly rigid block^10^. From another point of view the SSZ can be regarded as the metamorphic core of the larger Zagros accretionary complex which formed by the thickening of distal crustal segments of the Arabian margin^18,19^. The UDMA, which is situated between the SSZ and Central Iran, is thought to be mostly composed of Andean-type structures and is related to the magmatic activity from Eocene to the present^7,20^.

The ongoing collisional processes cause high seismicity in this region; the majority of earthquakes recorded in Iran occur in the Zagros. The strongest events, like the earthquake of Sare-Pol-Zahab on November 12, 2017 with magnitude 7.3 M_w_, represent a serious hazard to the population and infrastructure of densely populated areas of Iran.

The deep structure of the crust and mantle is a key element to understand the geodynamic framework of regional collisional processes. Many geophysical multiscale studies have been performed in the Zagros region based on different seismic and non-seismic data. Numerous studies based on receiver function analysis have provided generally consistent information about the crustal thickness beneath Zagros and surrounding areas^21–27^. Based on these studies, it can be concluded that the ZFTB has a crustal thickness of 45 ± 3 km, whereas beneath the SSZ, the Moho depth significantly increases up to $65\pm 3$ km. Similar conclusions follow from the teleseismic tomography study by Paul *et al*.^28^ who observed thickening of the crust in the SSZ (front of the MZT) and explained it as a signature of crustal doubling due to underthrusting the Arabian crust underneath Central Iran.

The mantle structures beneath the Zagros have been studied using regional tomography methods based of body and surface wave data^29–31^. Hafkenscheid *et al*.^32^ and Mohammadi *et al*.^33^ found a thick lithosphere beneath the Zagros which they considered as an indication of oceanic slab break-off in the Zagros collision zone. The regional travel time tomography studies by Alinaghi *et al*.^34^ and Koulakov^35^ revealed a subducting slab beneath Zagros turning to a nearly 90^0^ dip angle across the upper/lower mantle boundary.

Among the many geophysical studies of Zagros and surrounding areas, local earthquake tomography (LET), which uses travel time data of both stations and earthquakes located in the study area, has never been performed for the entire Zagros. This method, which provides information on seismic structures down to ~100 km depth, appears to be complementary to the previously derived results of regional tomography and receiver function analysis and can help us to answer some questions related to the lithospheric structure of the Zagros collision zone.

## Data and Methods

In this study, we use arrival time data from 2006 to 2018 which have been collected by the Iranian Seismological Center as well as International Institute of Earthquake Engineering and Seismology seismic networks. The dataset used for tomography consists of 123,575 P- and 11,520 S-picks from 7783 events with magnitude ${M}_{N}\ge 2.5$^36,37^. The distributions of earthquakes and stations used in the analysis are shown in Fig. 1B. The events are presented according to the different sizes of magnitude and depths in the figure. Histogram of data distribution with respect to the distance is depicted in Supplementary materials Figure S1. The distribution of the whole data as a function of magnitude and epicentral distance is plotted in the same figure.

We used the LOTOS code, Koulakov^38^, developed for simultaneous inversion for the 3D distributions of the P and S wave velocity anomalies and source locations. In the first step, LOTOS determines initial source locations using tabulated values of travel times previously calculated in a 1-D velocity model. The iterative algorithm of tomographic inversion includes the following steps: (1) Source relocations in the updated 3-D velocity structure based on the ray tracing bending method, (2) calculation of the first derivative matrix and (3) simultaneous inversion for P and S wave velocity anomalies, earthquake source parameters (4 parameters for each source), and station corrections. The inversion uses the LSQR method^39^. Further details regarding the data and inversion procedure are described in the Supplementary Information.

## Results and discussion

**Figure 2:**
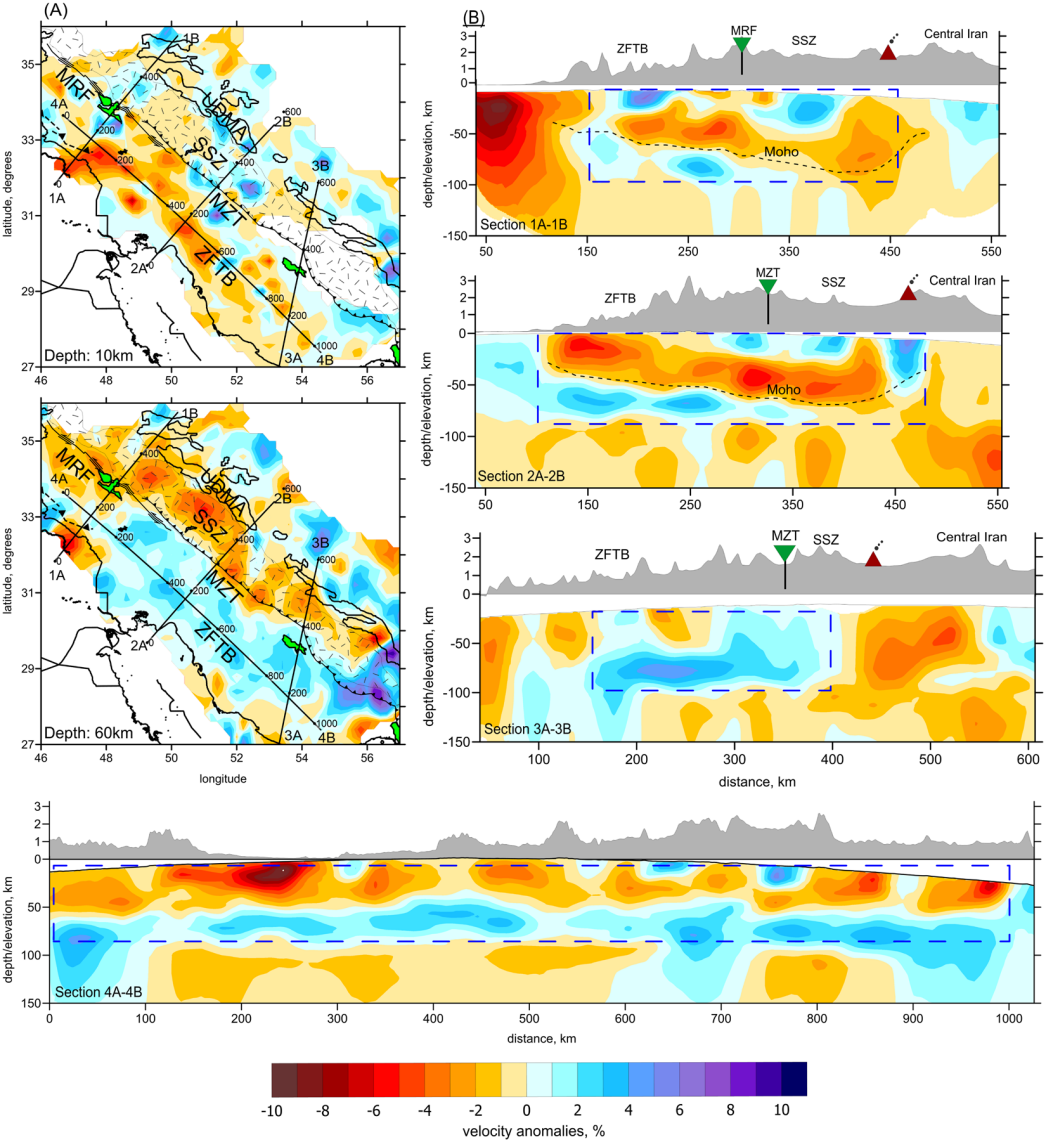
Results of tomography inversion. (**A**) Anomalies of P-velocity in two horizontal sections, derived from iterative tomography inversion. The main tectonic structures associated to the Zagros collision zone are plotted in all sections. Black lines plotted in horizontal sections indicate profiles used for showing the main results of vertical section. (**B**) P-velocity anomalies in four vertical sections. Dashed black line in section 2A-2B is a transition between low-velocity and high velocity anomalies which shows a good consistency with Moho depth variation that extracted from other studies. The well resolved areas are distinguished by blue dashed rectangle. Topography at exaggerated scale is presented above each section. Green reverse triangles represented the location of MRF and MZT in Zagros collision zone. Red triangles highlight the almost position of Urumieh-Dokhtar Magmatic Arc field. The figure was generated using the software Surfer (Version 13, http://www.goldensoftware.com/products/surfer).

The resulting 3-D distributions of the P wave velocity (Vp) anomalies are presented in two horizontal sections (Fig. 2A) and four vertical profiles (Fig. 2B). Further images corresponding to other horizontal sections, as well as the results for the S-wave velocity model, are presented in Supplementary materials Figures S6, S7 and S8. The Vp and Vs anomalies, which were obtained independently, appear to be almost identical in the crust (depths < 45 km). In deeper sections, the resolution of the Vs model is poorer due to considerably smaller number of the recorded S-waves travelling in the mantle and lower quality of the S-wave picks. For this reason, mantle structures are interpreted based only on the Vp model. To evaluate the spatial resolution of the tomography results, we have performed several synthetic tests, which are presented in the Supplementary Information in Figures S11, S12, S13, S14 and S15.

The most important structures related to the architecture of the colliding plates can be revealed in vertical sections in Fig. 2B. In section 1 and 2, we observe an elongated low-velocity anomaly, which is gently sloping to the northeastern direction in the P velocity model. Below and above, this anomaly is bounded by higher-velocity anomalies. It seems plausible to interpret this structure in terms of the subduction of the Arabian Plate underneath the Central Iranian Microplate. In this case, the low-velocity anomaly represents the crust and the underlying high-velocity anomaly would be associated with the mantle lithosphere of the Arabian Plate. In Fig. 2B, we show the boundary of these anomalies with a dashed black line which can be considered as Moho depth according to pervious receiver function studies^21–27^.

However there are several arguments against this interpretation. First, the regional mantle tomography models of Alinaghi *et al*.^35^ and Koulakov^35^ clearly show that the Arabian Plate deepens beneath the Zagros becoming nearly vertical. This seems to contradict the observation in our model of a nearly horizontal anomaly that propagates beneath the ZFTB and SSZ and almost reaches Central Iran. Second, the high-velocity anomaly located below the “crust-related” low-velocity anomaly appears to be narrower than would be expected if it was associated with the mantle lithosphere. This might be explained as a result of limited resolution of the tomographic inversion at this depth. However, the synthetic test with realistic configurations of anomalies (presented in Supplementary materials Figure S14) shows that a thick high-velocity layer representing the mantle lithosphere would be correctly recovered by the inversion. Therefore the resolution should be sufficient to discriminate between models with thick and thin layers at this depth, and we conclude that the actual high-velocity layer is thin and is unlikely to represent the mantle lithosphere. This is also supported by additional resolution tests in Figure S13 showing that at depths below 90 km, we still have resolution for large-scale anomalies.

**Figure 3:**
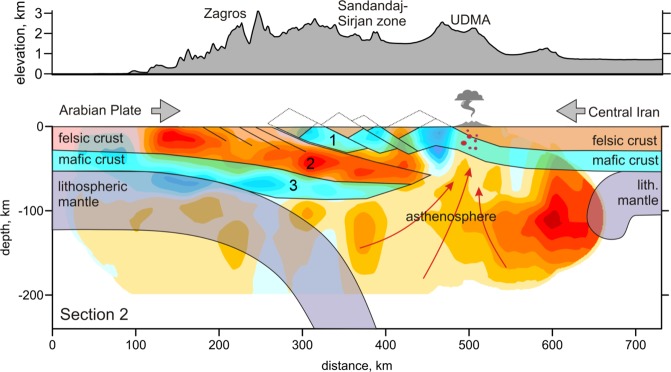
Conceptual scheme of the Zagros collision zone. The Vp distribution of section 2A-2B is used as the background velocity anomaly. In figure, number 1 represents the uplifted mafic layer of the Iranian plate’s crust as well as the felsic and mafic parts of the Arabian crust are represented by number 2 and 3, respectively. The figure was generated using the software Corel Draw (version X3; http://www.corel.com).

To explain the observed configurations of seismic anomalies in our model, we propose a scenario schematically illustrated in Fig. 3. We suggest that the low-velocity layer and the underlying narrow high-velocity anomaly represent the felsic and mafic parts of the Arabian crust, respectively. The overlying high-velocity anomalies can be interpreted as the uplifted mafic layer of the Iranian plate’s crust. According to this interpretation the lithospheric mantle of the Arabian Plate was detached from the crust. In our tomography result, the subducting mantle lithosphere cannot be revealed, and we draw it schematically in Fig. 3, taking into account the results of the regional tomography models by Alinaghi *et al*.^35^ and Koulakov^35^, in which the Arabian slab steeply subducts beneath Zagros.

We propose that these structures represent the ongoing process of relamination of the low-density Arabian continental crust, which was detached from the high-density mantle lithosphere and propagates underneath rheologically stronger crust of the Iranian plate. A similar scenario has been used to explain the presence of felsic channels presumed to exist in some modern and ancient continental collision zones, such as the Tibetan-Himalayan system or the Variscan orogeny^2,4,40^ as well as in some subduction zones^41^. Although this idea is supported by various geological and geophysical data the physical mechanisms and the dynamics of formation of such channels have been investigated so far only on the basis of numerical modeling^2,40^. We claim that in this study, we can provide for the first time a direct image of the relamination process consisting in forming a thick felsic rock channel beneath an entire orogen. In the Pamir, for instance, the detachment of felsic upper crust from the downgoing continental slab has been visualized tomographically^42^, but the material appears to not propagate horizontally under the orogen. In our case, the tomography results show an inverted crustal architecture of the collision zone, in which the low-velocity felsic (granitic and sedimentary) upper crustal rocks of Arabian plate form a narrow, seismically inactive^2,39^ lower crustal channel below the higher-velocity mafic (basaltic) middle-upper crustal layer of the thickening Iranian crust. Based on existing numerical models^2,40^, we suggest that the felsic channel formation is likely produced by separation (delamination) of the rheologically weak felsic upper crust of the subducting Arabian plate and its ductile propagating under rheologically stronger upper-middle crust of the Iranian plate. This intra-crustal relamination process is likely enabled by the hot thermal structure of the upper plate that creates conditions for rheological decoupling at the Moho^40^.

A shallow high-velocity anomaly beneath the SSZ can be interpreted as an exhumed mafic part of the Iranian crust. The fact that this shallow high-velocity anomaly looks non-continuous and seems to be separated in several parts can be explained by alternation of the uplifts and subsidence areas, as schematically illustrated in Fig. 3. This hypothesis is supported by the presence of large metamorphic complexes along the SSZ that could originate due to exhumation of the lower crust in uplift areas.

The interpretation of the Moho interface appears to be more complicated, as was suggested in previous studies. Doubled crust beneath the Zagros and SSZ causes an unusual structural inversion: the exhumed mafic crust of the central Iranian microplate is observed in the upper part, whereas felsic crust of the underthrusted Arabian Plate is dominant in the lower part. In Fig. 2B, we plot the Moho interface estimated in previous studies using receiver function^21–27^. A key structure for understanding the crust-mantle transition is the narrow high-velocity anomaly, which is marked with 3 in Fig. 3. If we assume that this high-velocity zone may represent the mafic part of the Arabian crust, as schematically shown in Fig. 3, the Moho depth would increase by at least 10 km compared to those obtained by other researchers who interpreted this transition from low to high seismic velocity as the crust-to-mantle transition.

Finally, beneath the UDMA at mantle depths, we observe prominent low-velocity anomalies. As was proposed for other similar collision zones, such as the Caucasus^43,44^, active crustal shortening causes delamination of the mantle lithosphere^45^. As a result, beneath the collision belts the mantle lithosphere layer is either strongly thinned or absent. The delaminated mantle lithosphere is then replaced by hot asthenosphere, which may appear directly beneath the crust and transport heat from the mantle to the surface. In turn, this may cause overheating of the crust and the formation of volcanic fields such as the UDMA in the Zagros collision zone.

## Conclusion

In this study, we have performed the travel time tomography for the entire Zagros using available data on regional seismicity. The derived models of Vp and Vs anomalies were carefully verified using a set of synthetic tests, as well as by independent inversions of separate data subsets (odd/even test).

The main feature resolved in the tomography model is a gently sloping inclined low-velocity anomaly that dips to the northeast. This anomaly is interpreted as the felsic crust of the continental Arabian Plate that propagates underneath the rheologically stronger crust of the central Iranian plate. This anomaly seems to be overlain by a high-velocity anomaly that may represent the exhumed mafic part of the Iranian crust. On the surface, this high-velocity anomaly coincides with metamorphic complexes within the Sanandaj–Sirjan Zone and ophiolites. The process of underplating of a silicic upper-crustal layer underneath the mafic lower crust during collision, called relamination, was previously presumed for some modern and ancient collision zones. In this study, we present a tomographic model that clearly images the crustal channeling beneath the Zagros.

Beneath the Zagros we observe a paradoxical situation: the felsic crust, which normally should be above, is located below the mafic crustal layer. This complex doubling of the crust makes it difficult to estimate the crustal thickness beneath the Zagros. We propose that the interface, which is interpreted as Moho in the existing receiver function studies, actually represents the boundary between the felsic and mafic crustal layers in the subducting Arabian Plate, and the actual crust-mantle interface is considerably deeper.

## Supplementary information

Supplementary Information.
