# Supplementary material for: Ongoing formation of felsic lower crustal channel by relamination in Zagros collision zone revealed from regional tomography
Source: Sci Rep. 2020 May 19;10:8224. doi: 10.1038/s41598-020-64946-w (PMC7237424; doi:10.1038/s41598-020-64946-w)
Supplement: Supplementary file 1 — Supplementary Information. [file 41598_2020_64946_MOESM1_ESM.docx]

**Ongoing formation of felsic lower crustal channel by relamination in Zagros collision zone revealed from regional tomography**

Supplementary Information

**Amir Talebi (1), Ivan Koulakov (2, 3), Ali Moradi* (1), Habib Rahimi (1), Taras Gerya (4)**

1. Institute of Geophysics; University of Tehran, Tehran, Iran.
2. Trofimuk Institute of Petroleum Geology and Geophysics, SB RAS, Prospekt Koptyuga, Novosibirsk 630090, Russia.
3. Novosibirsk State University, Novosibirsk, Russia, Pirogova 2, Novosibirsk 630090, Russia.
4. ETH Zurich, Dep. Of Earth Sciences, Sonneggstrasse 5, Zurich 8092, Switzerland

*Corresponding author: [asmoradi@ut.ac.ir](mailto:asmoradi@ut.ac.ir)

This material includes the description of data and algorithms. It also presents the results of the inversion of experimental data and synthetic tests.

Data and algorithms for inversion tomography

The data set used in this study consist of arrival times from regional seismicity in the time interval of 2006-2018 provided by Iranian Seismological Center, Institute of Geophysics, University of Tehran (IRSC, IGUT) as well as International Institute of Earthquake Engineering and Seismology (IIEES) of Iran. We have merged arrival time catalogues of both centers and created a uniform dataset. In total, the number of seismic stations that were employed in our seismic tomography was 67. For tomography, we applied basic selection criteria accepting only the events having the number of picks of more or equal 6. Thresholds of 1 s and 1.5 s were set for the absolute values of P and S time residuals, respectively. These values were estimated based on the expected size and amplitudes of seismic velocity anomalies in the study area.

After applying the criteria, the dataset used for simultaneous location of sources and tomographic inversion contained 123,575 P- and 11,520 S-picks from 7783 events with magnitude greater than 2.5. The average number of picks per event was 17.35. Such discrepancy between the numbers of the P and S picks is rather typical for most of regional and global databases (such as the ISC catalogue) as the national agencies usually provide the first arrivals and very few later phases.

Figure S1 shows the histogram of data distribution with respect to the distance. Distribution of data as a function of magnitude and epicentral distance is presented in the same figure. All these events have the magnitudes greater than 2.5 (see Figure S1). Most of them correspond to the magnitude interval between 3.6 – 4.6. The P-waves arrivals from such events are clearly detectable and picked with high accuracy. The data agencies does not provide an explicit estimate for the picking accuracy. Based on examination of a significant number of seismograms, we estimate the accuracy of the first arrival time determination at ~0.15 s.

It can be seen in Figure S1 that most of data correspond to epicentral distances of up to 1000 km, which is enough to illuminate the uppermost mantle. In the inversion, we used both crustal (Pg, Sg) and mantle (Pn, Sn) phases. However, in the input file for tomography, there is no distinguishing of these two cases. When locating events, if there are several rays connecting the source and receivers, the algorithm select one providing the minimum residual.

The distributions of the P and S wave raypaths are presented in different depth intervals (Figure S2), as well as along vertical sections (Figures S3 and S4), same as used to present the results of the tomographic inversion. These plots give us the first view to the resolution that can be expected from this dataset. Further estimates are made with the use of synthetic modeling presented later.

The seismic tomography was carried out using the LOTOS code developed by Koulakov^1^. For our study, we used a version, in which the sphericity of Earth was taken into consideration because of the large size of the area.

The proper selection of an initial reference model plays an important role in seismic tomography. Some artifacts can be projected into final results due to an inaccurate reference velocity model. Some local 1-D velocity models have been presented for different areas in Zagros collision zone^2,3,4,5,6^. Hence, different 1-D velocity model were tested to find the best initial seismic velocity model. Three different 1-D velocity model are shown in Table S1. The shear wave velocity was driven from the results of Motaghi et al^6^. They estimated a 2-D shear velocity model for the crust along a profile which crossed the Zagros collision Zone. The constant $\frac{V_{P}}{V_{S}}$ ratio was defined equal to 1.75. As seen in Table S2, after source location in the first iteration, the model (Ref_03) that is the combination of both Yaminifard et al^5^ and Motaghi et al^6^ leads to smaller average deviation of residuals in comparison of those models reported by other studies. The number of data selected after source location are presented in same Table, and it can be found that Ref_03 again obtains larger numbers of events and picks. Based on the applied constrain, the Ref_03 was chosen as the best reference velocity model to start the tomography procedure.

Further estimates for the optimal 1D distributions of the P and S wave velocities in the reference model were based of iterative performing a series of the full tomography inversion cycles. Each time of running the program, the starting 1-D velocity model (Ref_03) was updated according to the average velocity derived in the previous run, which finally provided balanced amount of positive (blue) and negative (red) anomalies in the tomography model. As a result, a first optimizeed 1-D velocity model of crust (Ref_Zagros) has been obtained for the entire Zagros collision zone, which is more suitable for further tomographic inversion. The values of velocities at several depth levels according to the 1D reference model used for the tomography (Ref_Zagros) are reported in Table S3. Between these levels, the velocity values are linearly interpolated. In this model, we can distinguish a ∼10 km thick sedimentary (V_p_ ∼4.90 km s^-1^), the upper crust down to ∼30 km (V_p_ ∼ 5.54 km s^-1^) and the lower crust down to ∼45 km (V_p_ ∼6.30 km s^-1^). Our 1D velocity model is defined to provide continuous velocity anomalies and it can not be affected by any sharp interfaces and even high gradient levels. Hence, the Moho is not defined as a sharp first-order interface with variable depth and it was derived based on velocity anomalies.

The velocity distributions are parameterized by a set of nodes distributed inside the study volume. To avoid any artifacts related to predefined parameters of the grid we performed the inversions for four different grids with different basic orientations (0, 22, 45 and 66 degrees). For instance, Figure S5 shows node distributions of two grids with basic orientations of 0◦ and 45◦. The results with different basic orientation grids are averaged into one model. Note that, in map view, the nodes are distributed regularly with the spacing of 10 km. However, in the vertical direction, the number of nodes and spacing depend on the data coverage, but the distance between the nodes cannot be less than 5 km.

We used two types of regularization that balances the trade-off between model stability and resolution. The amplitude damping was performed by adding a diagonal matrix with zero data vector. The flattening of the model was controlled by another matrix, each line of which contained two nonzero elements with equal values, but opposite signs, that minimized the difference of velocity anomalies between all pairs of neighboring nodes. The optimal values of the inversion parameters used for tomography (Table S4) were estimated using the synthetic modeling to ensure the best recovery quality.

Inversion of experimental data

Based on the experimental data, we performed five iterations and used the same inversion parameters reported in Table S4. In the main paper, we present the P-wave velocity anomalies in two horizontal sections and four vertical sections. The distributions of the *Vp* anomalies for the further horizontal sections are shown in Figure S6. The results of *Vs* anomalies are presented in six horizontal and four vertical sections Figures S7 and S8, respectively. The absolute values of the Vp and Vs are shown in the same vertical sections in Figures S9 and S10, however, such representation appears to be less informative than the relative anomalies, and we do not use them for our interpretation.

The values of average residuals for the P and S wave data during the iterative inversion procedure are presented in Table S5. We can see that the P-wave residuals reduce from 0.41 s to 0.32 s (21% of variance reduction), whereas the S-wave residuals reduce from 0.72 s to 0.51 s (28.42% of variance reduction).

It can be found that the distributions of the Vp and Vs anomalies have similar configurations, especially for shallow depths. As the major regional structures usually affect similarly the P and S waves, this similarity of the P and S wave model can serve as a primary argument showing the robustness of the results. Moreover, different geological units in Zagros collision zone such as ZFTB, SSZ and UDMA are similarly resolved in the both P and S velocity perturbations.

Synthetic modeling

To examine the robustness of the solution, we have performed several synthetic tests. Furthermore, synthetic tests allow defining the optimal values of the inversion parameters such as damping and smoothing, which can be utilized in the case of the experimental data inversion.

In all cases, we produced a synthetic velocity model as a sum of the 1D reference model and anomalies defined by different algorithms. The synthetic travel times were calculated in the 3D synthetic model for the same source-receiver pairs as in the experimental dataset using the bending method of ray tracing. To provide more realistic conditions, synthetic travel times were perturbed by a random noise, which has an average deviation of 0.1 s for the P and 0.2 s for the S wave data. This noise level is slightly smaller than the picking error estimated from the seismogram examination (e.g., 0.15 s for the P-wave arrival). At the same time, this level provided a similar value of variance reduction (~20-30 %) in synthetic modeling as in the case of experimental data inversion, which indicates that this value is adequate. After computing the synthetic data, we “forgot” about source coordinates and origin times. The recovering of the model follows the same workflow as in the case of experimental data including the stage of the initial source location, which strongly biases the coordinates in respect to their true values. The tomographic inversion is performed using the same steps and same parameters as in the case of experimental data processing.

In Figure S11, we present the result of checkerboard tests aimed in assessing the horizontal resolution. In this test, the anomalies have the lateral size of 100x100 km size and remain unchanged with depth. The amplitudes of the P and S wave velocity deviations are ±5%. We used a synthetic model with anomalies having the lateral size of 100 km. The results of recovery of this synthetic model (Figure S11) show that in areas with sufficient amount of data and stations (central part of the study area), the checkerboard anomalies can be correctly restored. Fortunately for us, the most attractive area regarding to tectonically aspects (MZT and SSZ) coincided with the area of highest resolution. The resolution of the S-wave model appears to be similar to that of the P-wave model in the crust (down to 45 km depth), but in deeper sections, the Vs anomalies appear to be strongly smeared.

Another important question relates to the capacity of the tomography inversion to discriminate crustal and mantle structures. To investigate it, we have produced another checkerboard model, which change the sign of anomalies at 40 km depth (Figure S12). The recovery result shows that the best resolution is achieved for the P-wave model in the mantle and much poorer in the crust (only in areas with sufficiently large and dense seismic networks). For the S-wave data, this test shows sufficient resolution neither in the crust, nor in the mantle.

The vertical resolution is investigated using a series of tests shown in Figure S13. In all cases, the checkerboard anomalies were defined along the vertical section 4 passing along Zagros in the NW-SE direction. In Model 1, we consider a checkerboard with the lateral size of anomalies of 80 km and changes of sign at 20 and 80 km. It can be seen that the upper two layers of anomalies are clearly recovered, but the deeper structures are almost not resolvable. To further explore the resolution solely for the mantle structures, we defined two other models with anomalies starting from 60 km and 90 km (Models 2 and 3 in Figure S13, respectively). It can be seen that when the upper limit of anomalies is at 60 km depth, they are clearly resolvable, whereas for the model with anomalies located below 90 km depth, the recovered structures considerably lose the amplitude. At the same time, when we defined larger anomalies at the same depths (200 km instead of 80 km in Model 4), they are robustly resolvable. For the S-wave data, no mantle structures below 60 km depth can be recovered. All these tests show the realistic limitations of seismic tomography with the existing rays to recover the structures of different size and at different depth, which should be taken into account when considering the results of experimental data inversion.

In order to check the capacity of the tomography inversion to resolve the structures of realistic shapes, we created another synthetic model with polygonal anomalies defined in the vertical sections 1A-1B and 2A-2B, same as used for presenting the main results. The shapes of anomalies in this synthetic model were set similar to those observed after the inversion of experimental data (Figure S14, 1^nd^ row). The reconstruction anomalies (Figure S14, 2^nd^ and 3^nd^ rows) confirm that the shapes and patterns of main features are generally consistent with the real cases for both P and S model.

In order to assess the contribution of random noise in experimental data to the final tomography models, we performed inversions for two independent data subsets. In this test, all the data were divided into two equal datasets that included the travel times from events having odd and even numbers. The inversions, for these two data sets, were carried out independently with the same parameters and using the same scheme as in the case of inverting of the complete data set. In Figure S15, the results of inversion for P and S models corresponding to odd and even events can be compared with each other, as well as with the result based on the entire dataset. The results demonstrate practically identical anomalies in both P and S wave models at the crustal depths, suggesting fairly low effect of noise in the data on the inversion result. In the mantle, the Vp anomalies remain highly consistent, whereas, the Vs anomalies demonstrate some differences, which shows significant noise effect in this case.

Another concern relates to using the data from events located outside the station networks. To select data for tomography, many authors use the criterion of GAP<180 presuming that the sources should be located strictly within the station perimeter (the maximal azimuthal gap should not exceed 180 degrees). It is true that the accuracy of locations of out-of-network sources is lower in most cases due to the trade-off between source parameters and velocity distributions. However, such events give valuable information about relative time variations at stations, which can be used to resolve velocity anomalies beneath the network. It was shown in some studies^7^ that including out-of-network events greatly improves the variability of ray orientations in the study area and thus enhances the quality of tomographic imaging. Using only events having GAP<180 degrees in most cases strongly decreases the number of data and worsens the spatial resolution.

To access the effect of this selection criterion, we have produced another model only using the events with GAP<180 degrees (Figure S16, upper row). It can be compared with the main results obtained without implementing any GAP criteria (Figure S16, lower row). This selection strongly reduced the number of events almost twice, mostly because of rejecting a large number of events in the Makran zone in southeastern part of the study area. However, this reduction almost did not affect the tomography results in most part of the study area. It shows that in this particular case, implementing this selection criterion does not affect the results.

The final concern relates to the influence of the starting reference model to the shape of the recovered anomalies, particularly, to the appearance of the quasi-horizontal layer observable in the vertical sections. To access this effect, we have performed a series of inversions with experimental data using several different reference models. In Figure S17, we present the resulting P-wave velocity anomalies in Section 2 based on three different reference models. In these models, we varied the velocities in the upper and lower parts enabling different gradients of velocity increase with depth (see right panels in Figure S17). The case of the medium gradient corresponds to the main model, in which balanced distributions of positive and negative anomalies achieved at all depths. In the case of lower gradient (upper row in Figure S17), we obtained mostly “red” anomalies in the upper part and “blue” anomalies in the lower part of the model. In the case of the higher velocity gradient (lower row in Figure S17), we obtained the opposite patterns of the anomaly distributions: positive shallow and negative deep anomalies. Nevertheless, despite the considerable differences between these models, we see that the quasi-horizontal high-velocity channel at 60-70 km depth is observable in all cases. It means that this anomaly, which is used for our interpretation, is not related to inappropriate definition of the reference model and appears to be a robust feature.

**References for supplementary information:**

1. Koulakov, I. LOTOS code for local earthquake tomographic inversion. Benchmarks for testing tomographic algorithms. *Bulletin of the Seismological Society of America*. **99**, 194–214 (2009).
2. Hatzfeld, D., Tatar, M., Priestley, K. & Ghafory-Ashtiany, M. Seismological constraints on the crustal structure beneath the Zagros Mountain belt (Iran). *Geophysical Journal International*. **155**, 403–410 (2003).
3. Tatar, M., Hatzfeld, D. & Ghafori-Ashtiany, M. Tectonics of the Central Zagros (Iran) deduced from microearthquake seismicity. *Geophysical Journal International*. **156**, 255–266 (2004).
4. Nissen, E., Tatar, M., Jackson, J.A. & Allen, M.B. New views on earthquake faulting in the Zagros fold-and-thrust belt of Iran *Geophysical Journal International*. **186(3)**, 928–944 (2011).
5. Yaminifard, F., Hassanpour Sedghi, M., Gholamzadeh, A., Tatar, M. & Hessami, K. Active faulting of the southeastern-most Zagros (Iran): Microearthquake seismicity and crustal structure. *Journal of Geodynamics*. **55**, 56-65 (2012).
6. Motaghi, K. & Shabanian, E. Underplating along the northern portion of the Zagros suture zone, Iran. *Geophysical Journal International*. **210**, 375–389 (2017).
7. Koulakov I., Out-of-network events can be of great importance for improving results of local earthquake tomography. *Bulletin of the Seismological Society of America*. **99, 4,** 2556–2563 (2009).


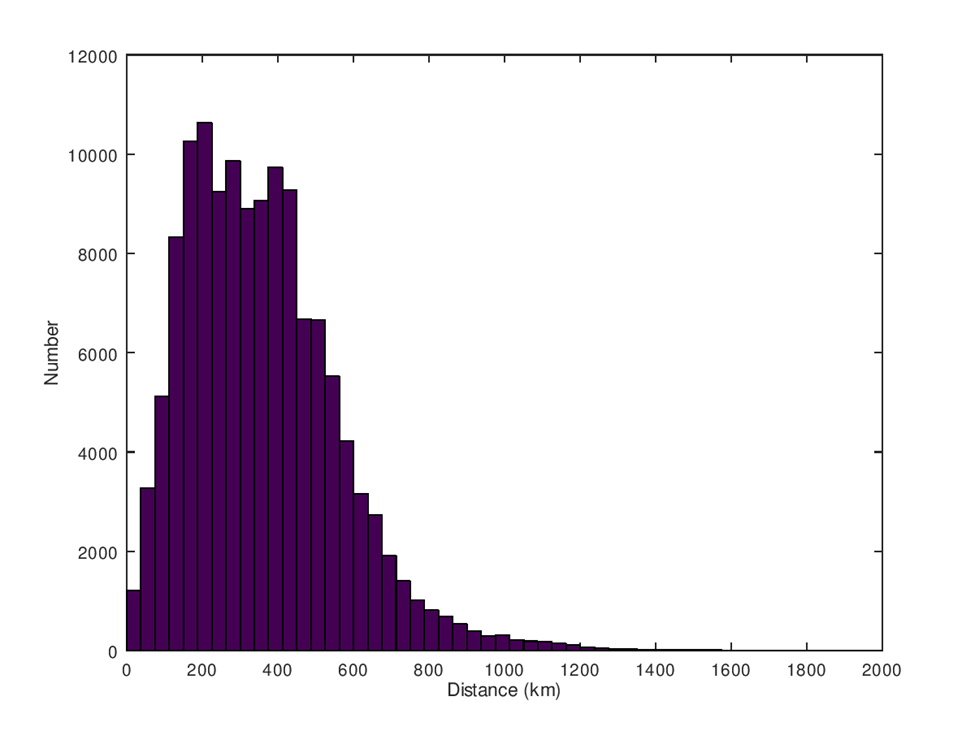


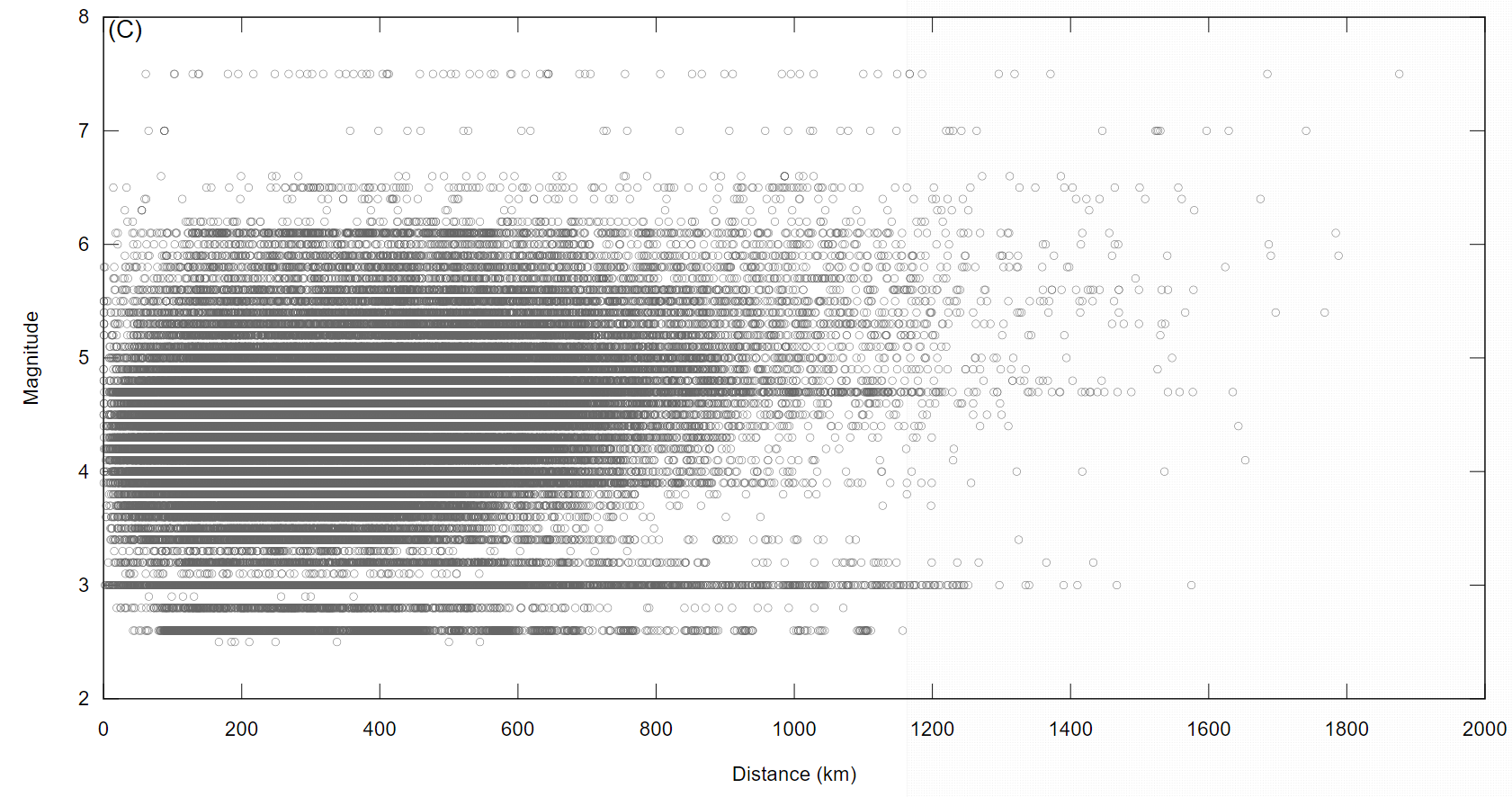


Figure S1. Histogram of data distribution with respect to the distance (top). Distribution of the whole data as a function of magnitude and epicentral distance (bottom).


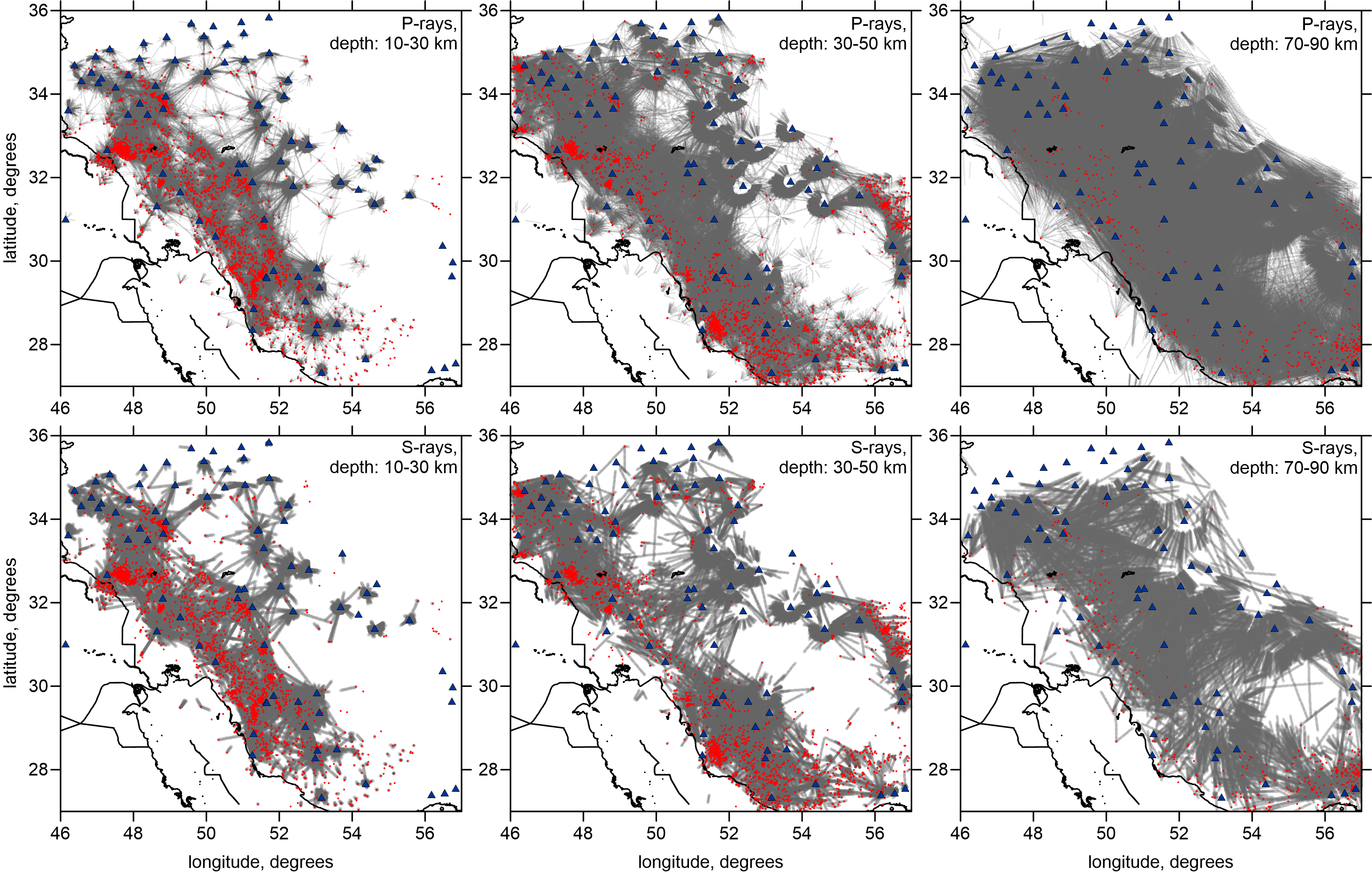


Figure S2. Ray distributions of the P and S waves (grey dots) in different depth intervals. Red points are the epicenters of earthquakes in the corresponding intervals, and the blue triangles are the seismic stations that contributed into the dataset used for tomography. The figure was generated using the software Surfer (version 13, <http://www.goldensoftware.com/products/surfer>).


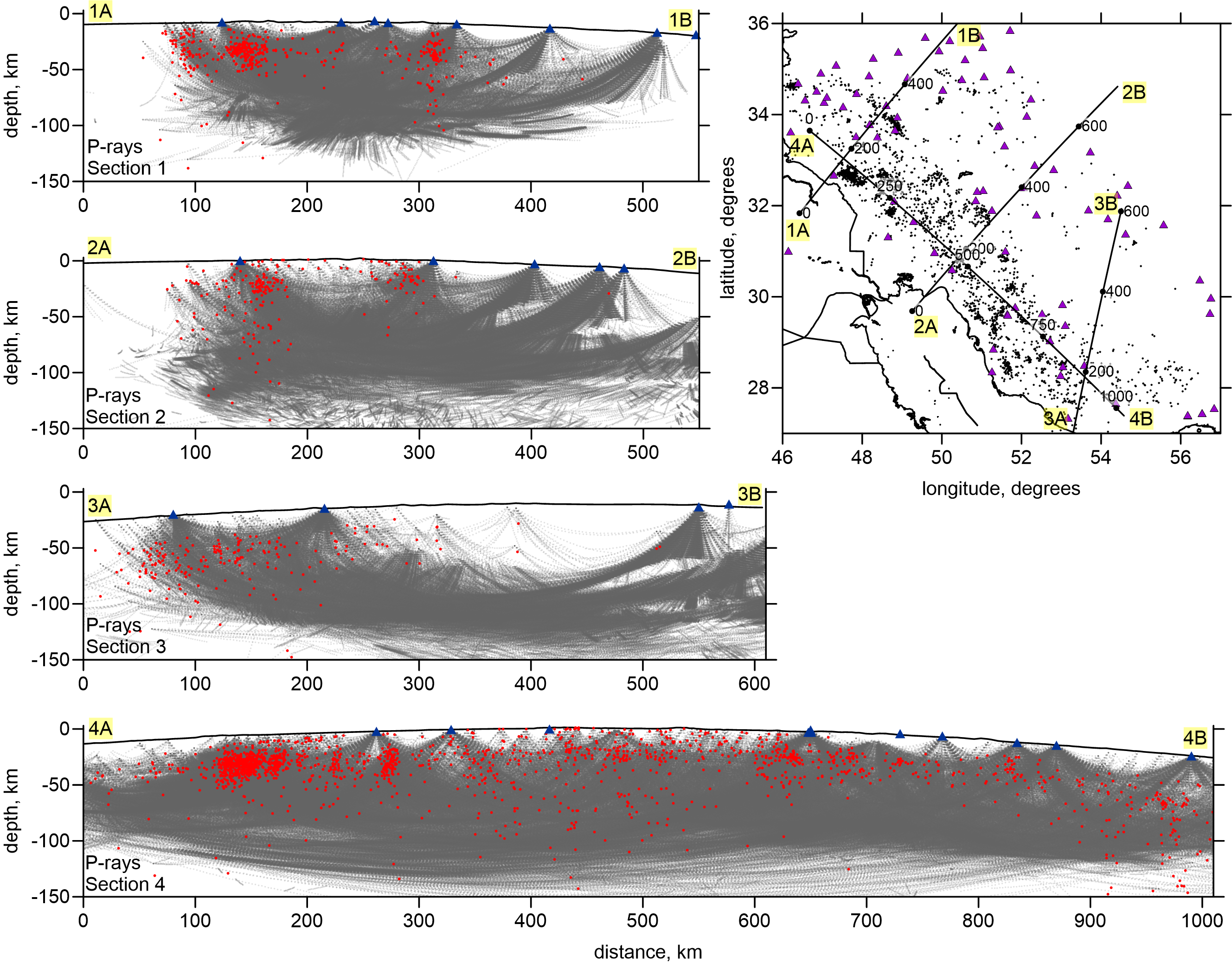


Figure S3. Distributions of the P-wave data along four vertical profiles. Rays (grey dots), events (red dots) and seismic stations (blue triangles) within a zone of 60 km width are projected to the profile planes. Locations of the profiles are indicated in the map.


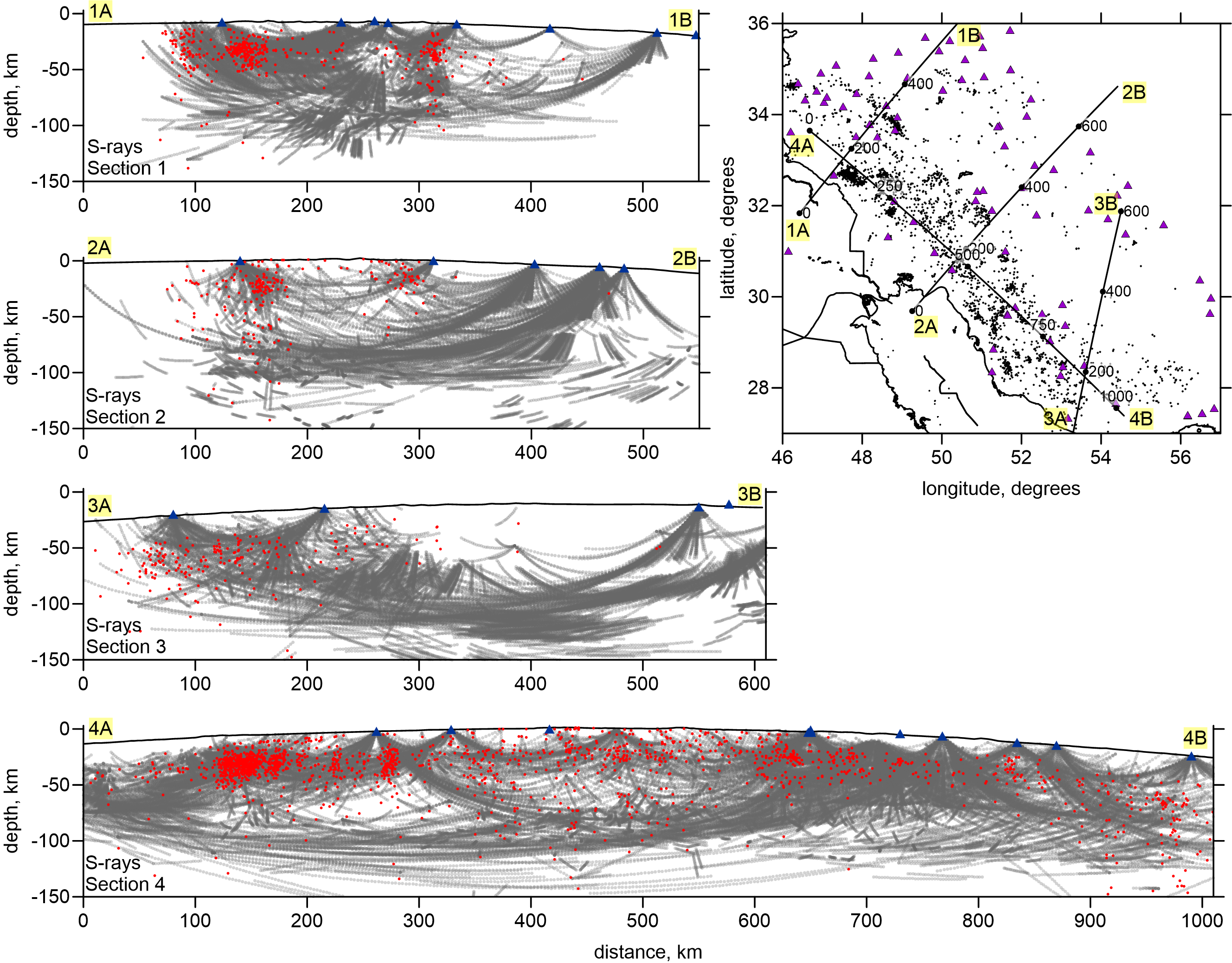


Figure S4. Same as Figure S4, but for the S-wave data.


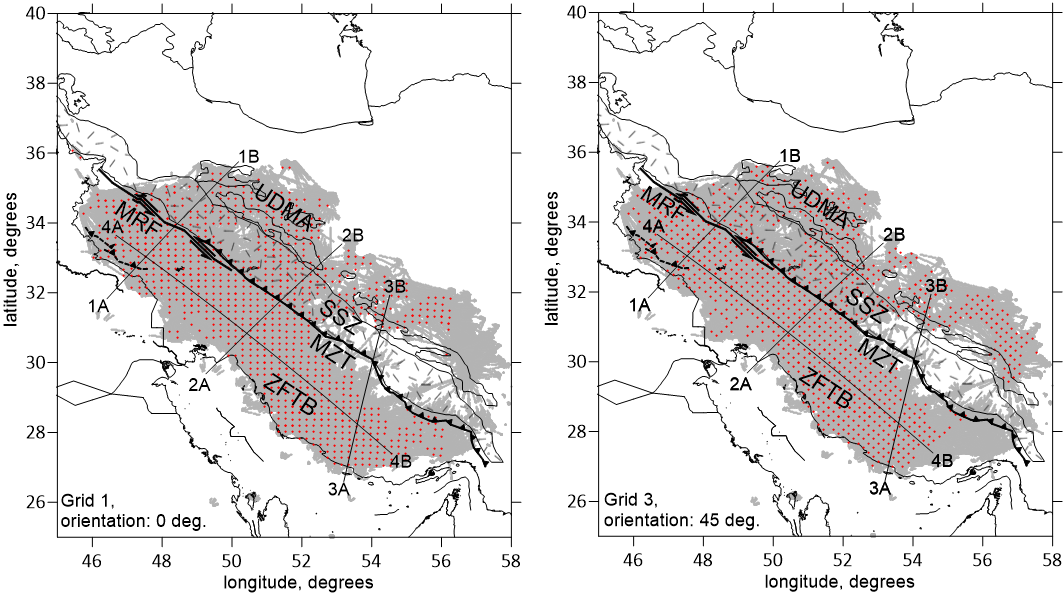


Figure S5. Distribution of P rays and two parameterization grids corresponding to basic orientations of 0 and 45 degrees. Red dots represent the location of each node. Four vertical cross section used in this study are shown by solid black lines. The main geological structures given for reference are the same as in Figure 1 of the main article. The figure was generated using the software Surfer (version 13, <http://www.goldensoftware.com/products/surfer>).


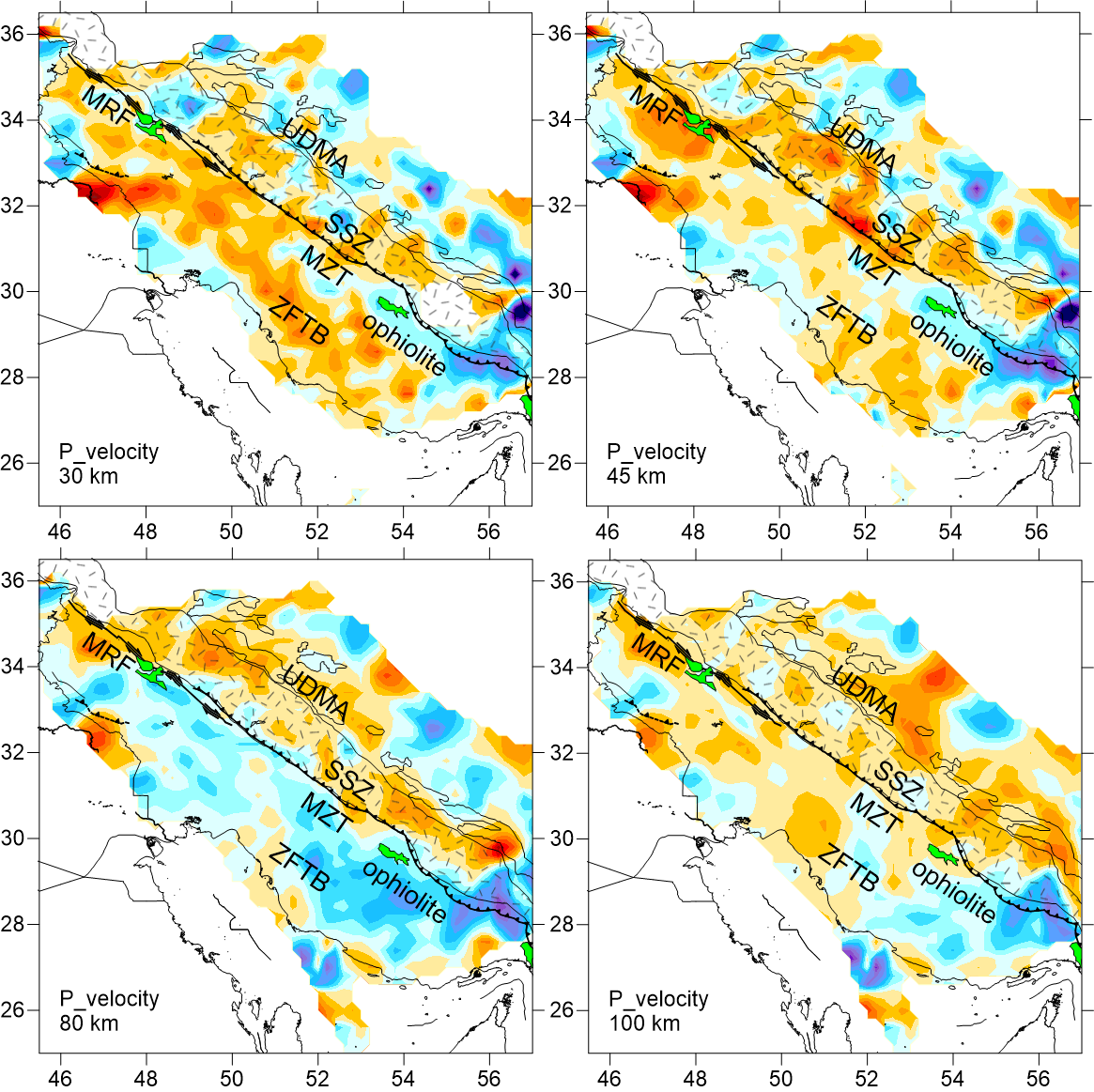


Figure S6. The *Vp* distribution of four horizontal sections. The main geological structures given for reference are the same as in Figure 1 of the main article. The figure was generated using the software Surfer (version 13, <http://www.goldensoftware.com/products/surfer>).


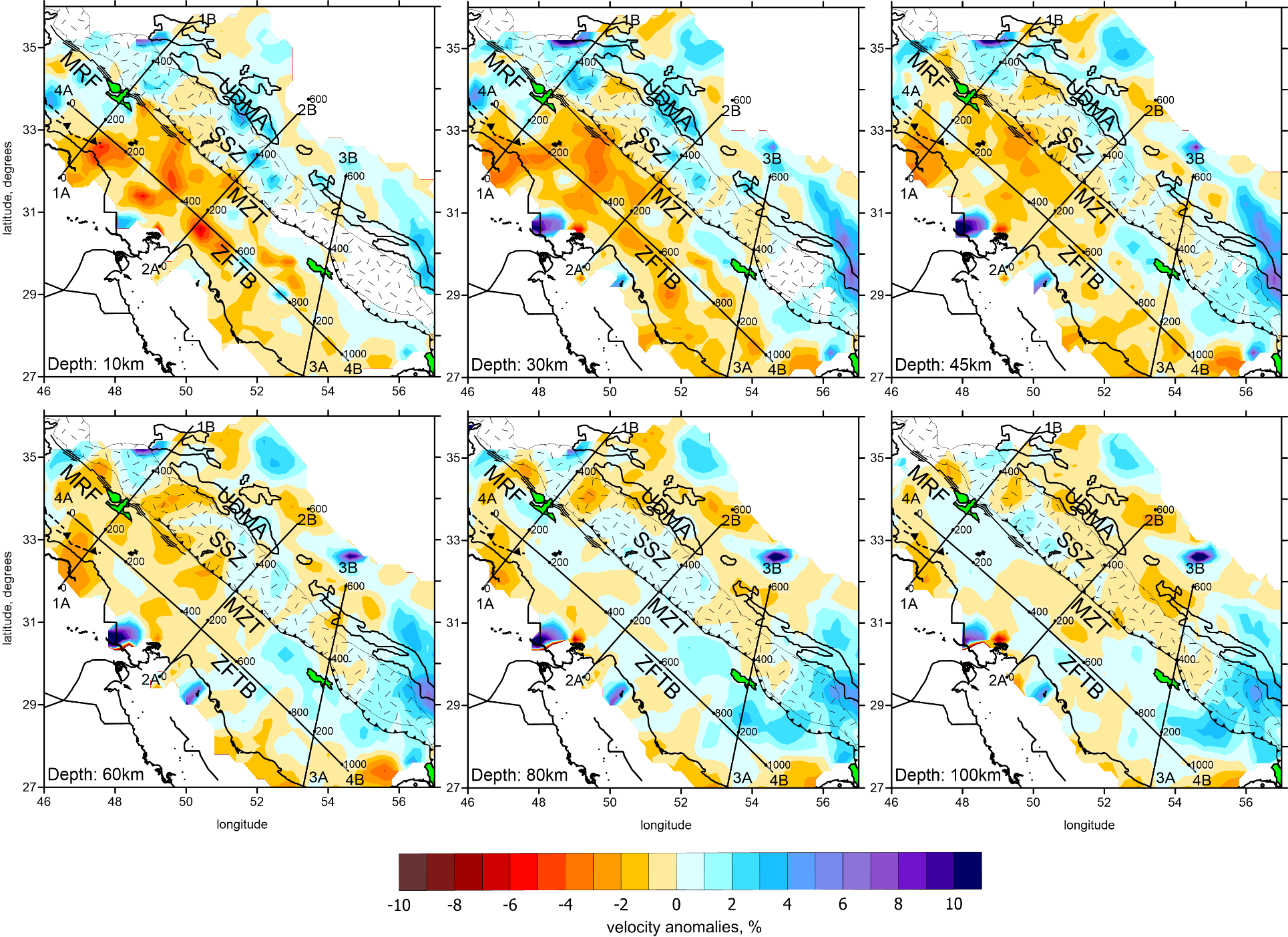


Figure S7. The results of Vs anomalies in six horizontal sections. The main geological structures given for reference are the same as in Figure 1 of the main article.The locations of the profiles related to the vertical sections are shown on the map. The figure was generated using the software Surfer (version 13, <http://www.goldensoftware.com/products/surfer>).


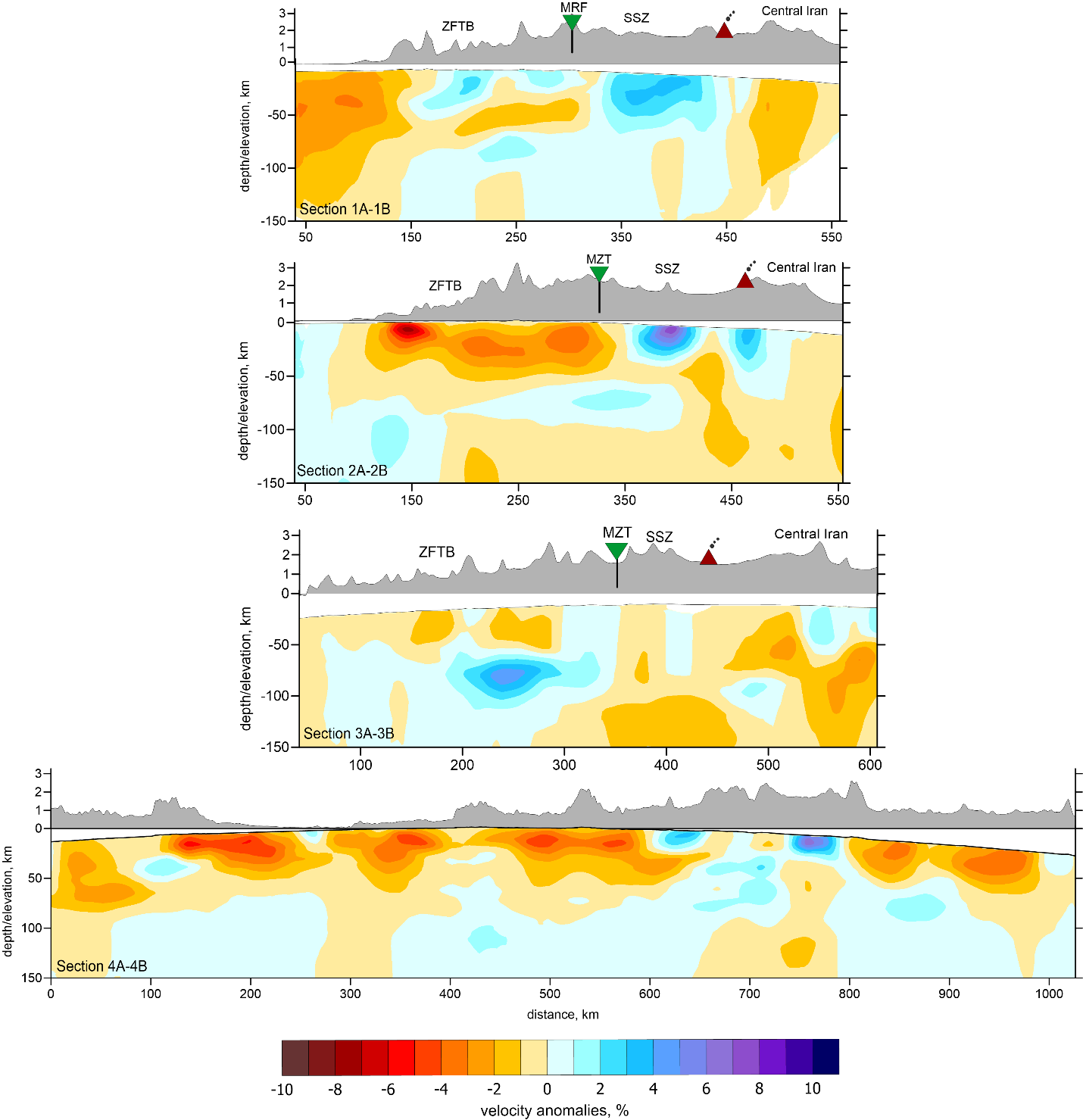


Figure S8. The results of *Vs* anomalies in four vertical sections. Locations of the profiles are shown in Figure S5.


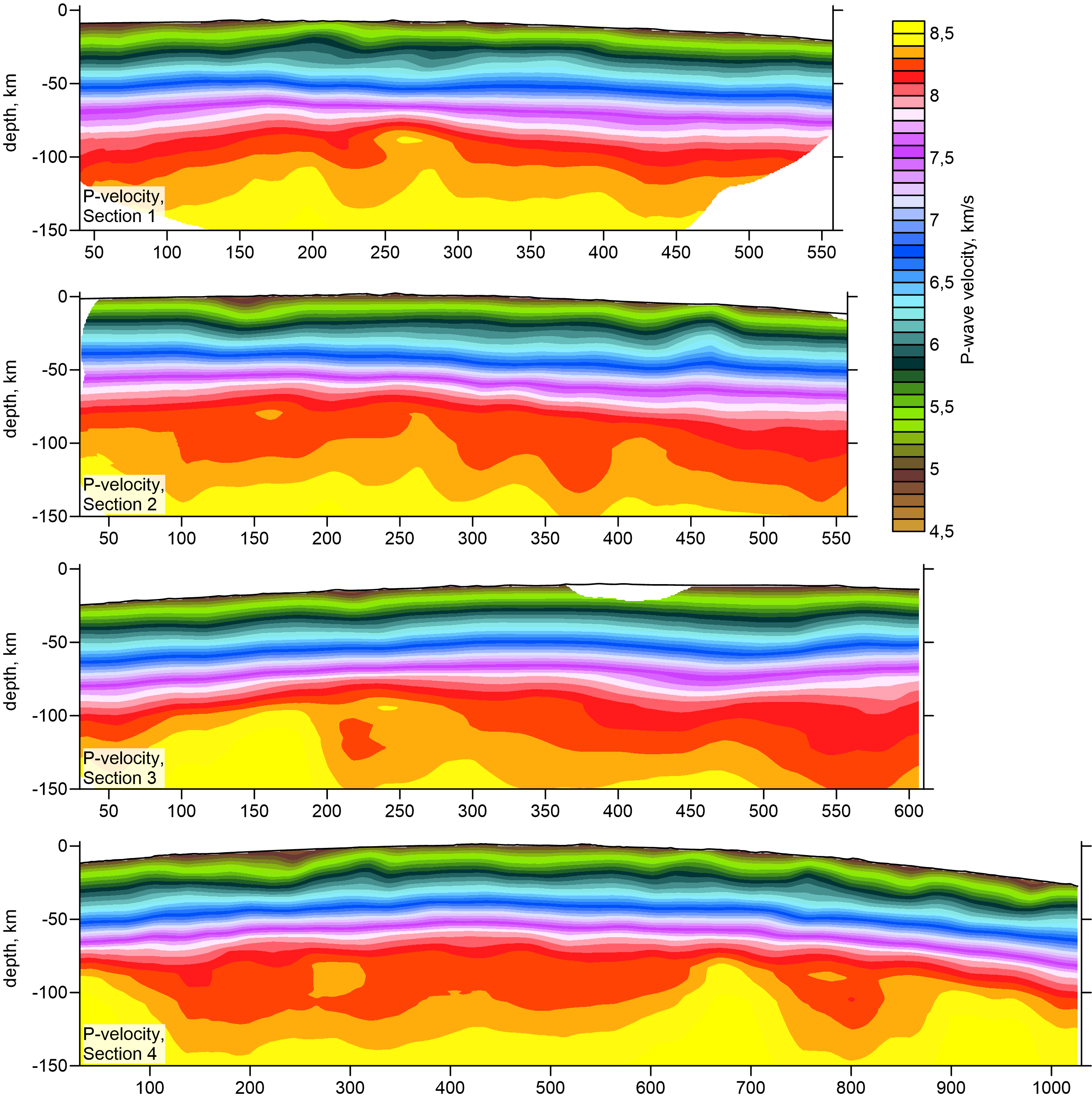


Figure S9. Absolute P-wave velocities in four vertical sections. Locations of the profiles are shown in Figure S5.


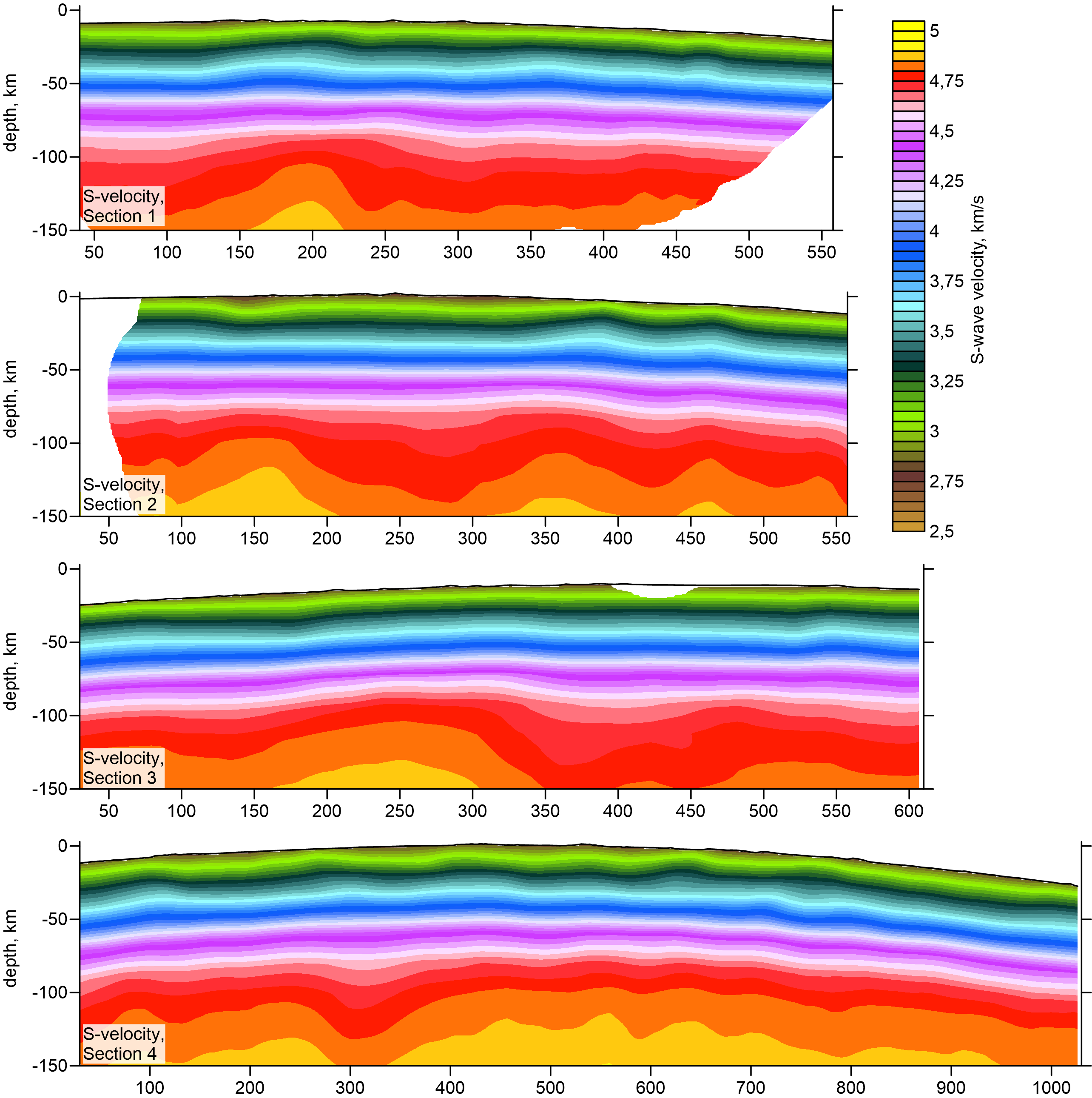


Figure S10. Absolute S-wave velocities in four vertical sections. Locations of the profiles are shown in Figure S5.


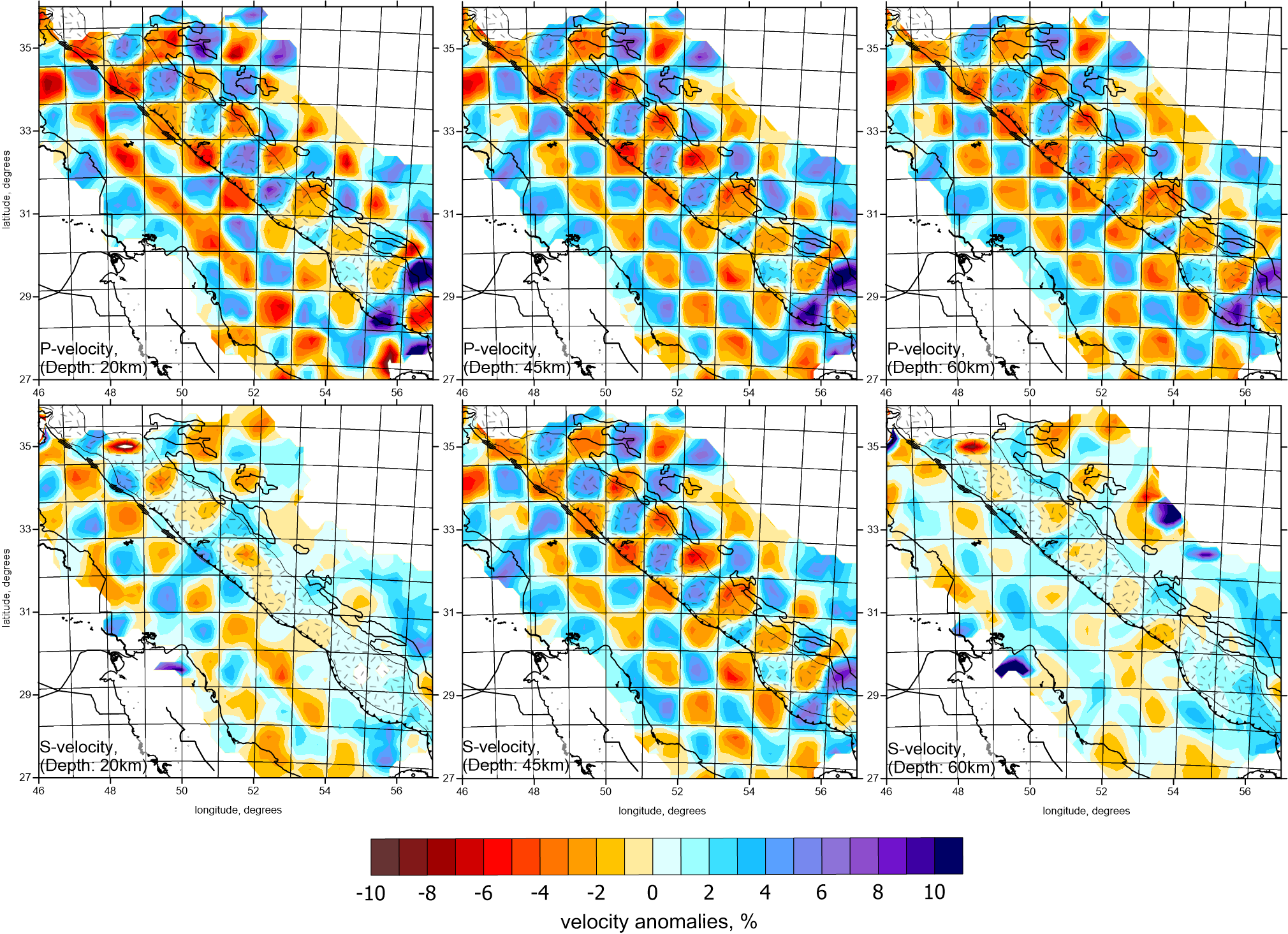


Figure S11. Checkerboard test with synthetic anomalies to examine the horizontal resolution. In the model, the anomalies remain unchanged in all depths. The shapes of the initial synthetic anomalies are highlighted with thin black lines. The results are presented at 20, 45 and 60 km depth for the P and S wave velocity anomalies. The main geological structures given for reference are the same as in Figure 1 of the main article. The figure was generated using the software Surfer (version 13, <http://www.goldensoftware.com/products/surfer>).


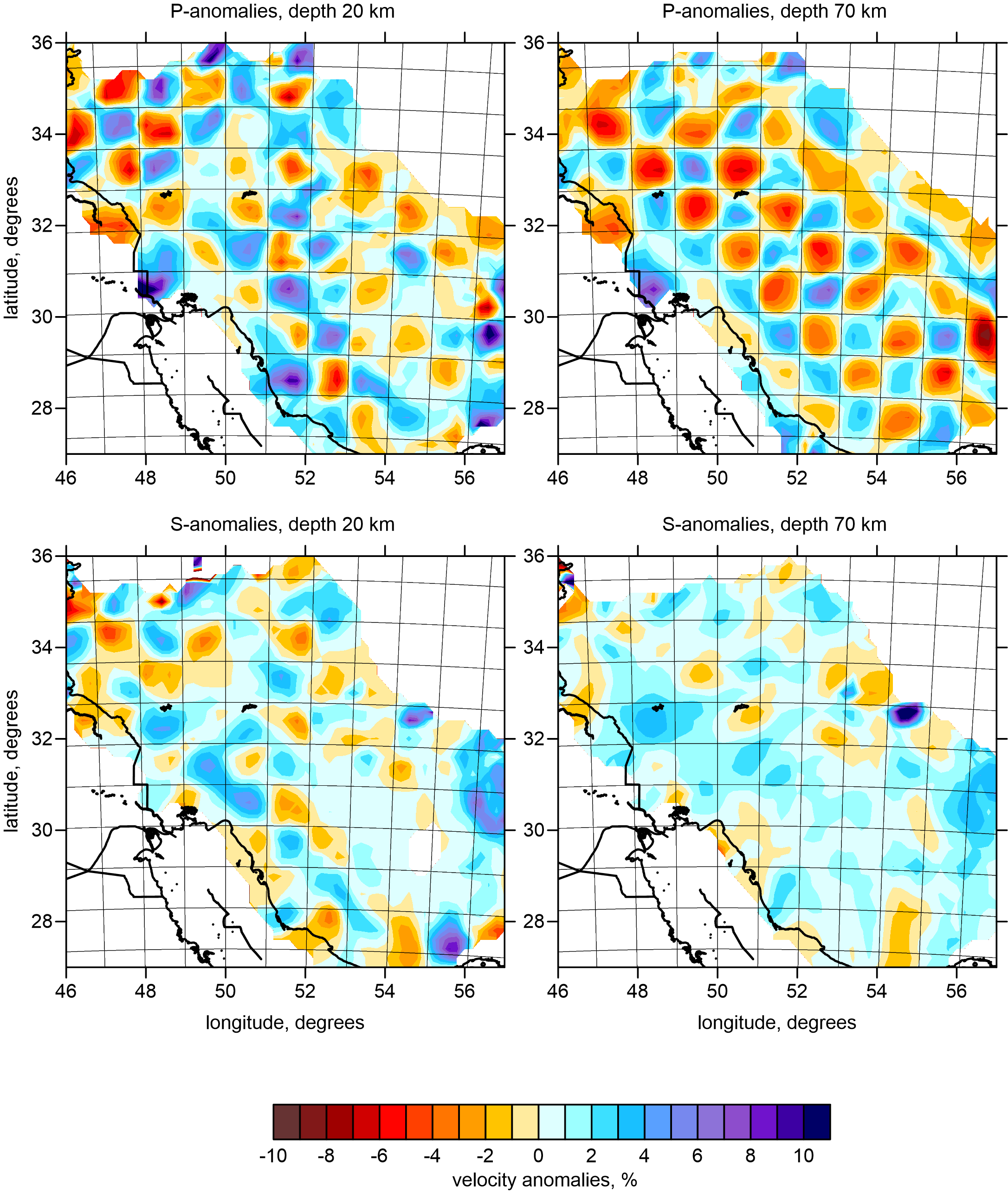


Figure S12. Checkerboard test for the two-layered model, in which the sign of anomalies changes at 40 km depth. The shapes of the initial synthetic anomalies are highlighted with thin black lines. It can be seen that for the Vp anomalies, the structures are resolved correctly, whereas, the test for the Vs anomalies fails. The figure was generated using the software Surfer (version 13, <http://www.goldensoftware.com/products/surfer>).


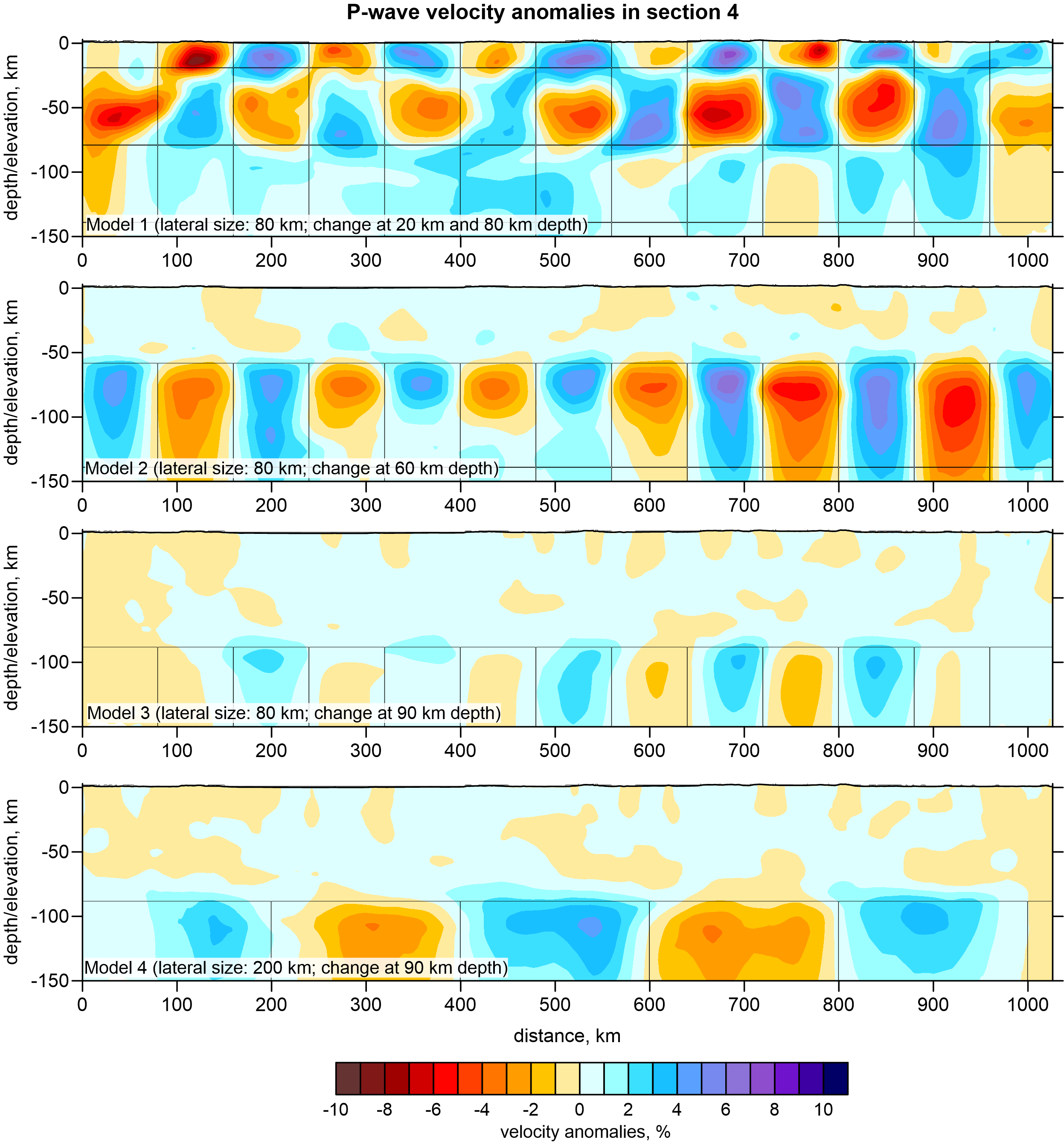


Figure S13. P-wave velocity anomalies resulted from a series of four synthetic tests to examine the vertical resolution along the vertical section 4. The shapes of the synthetic anomalies are highlighted with thin black lines. The parameters of the anomalies are indicated in each plot.


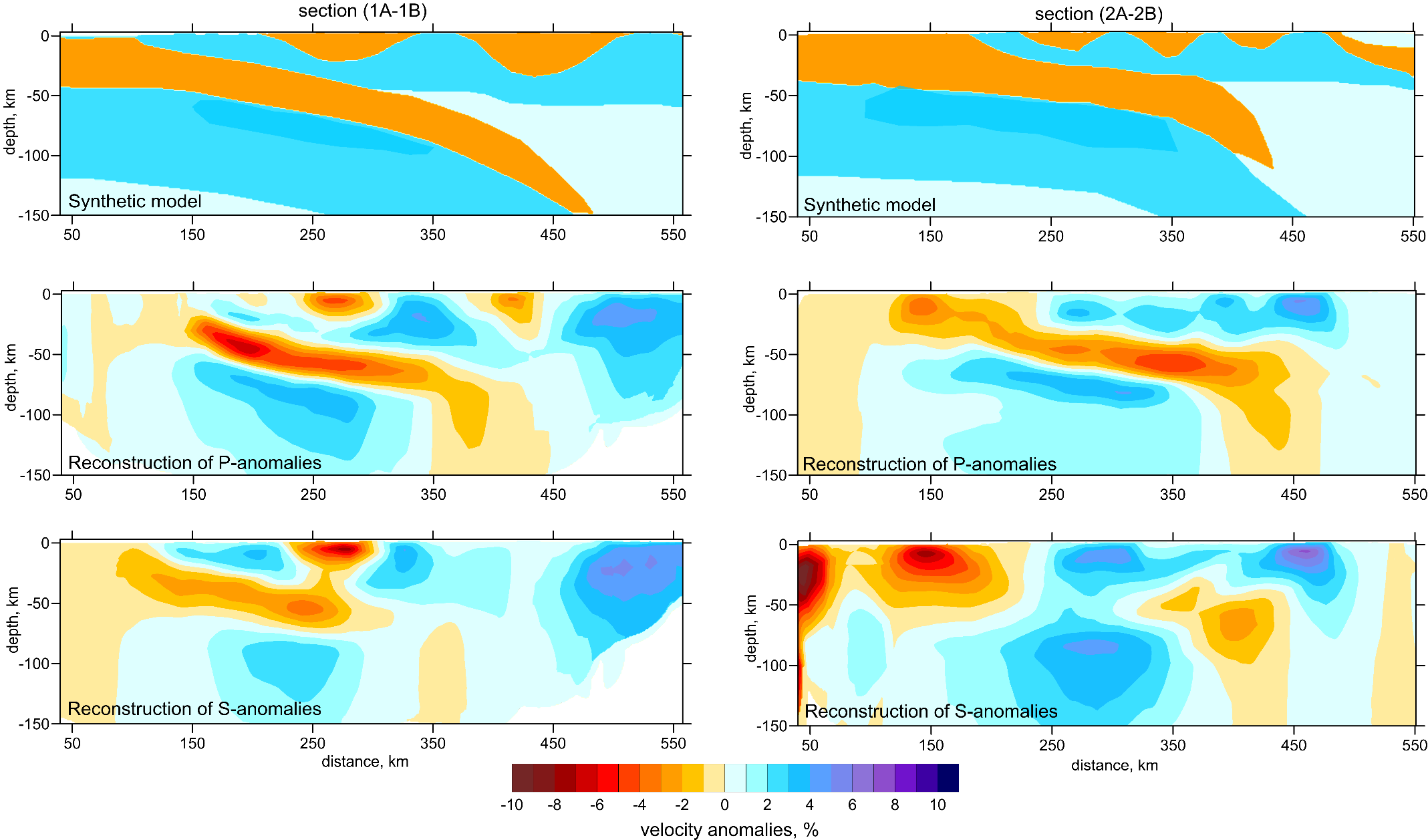


Figure S14: Synthetic tests with realistic patterns defined in two vertical sections (1nd row). The 2nd and 3nd rows correspond to the reconstruction results for the P and S anomalies, respectively.


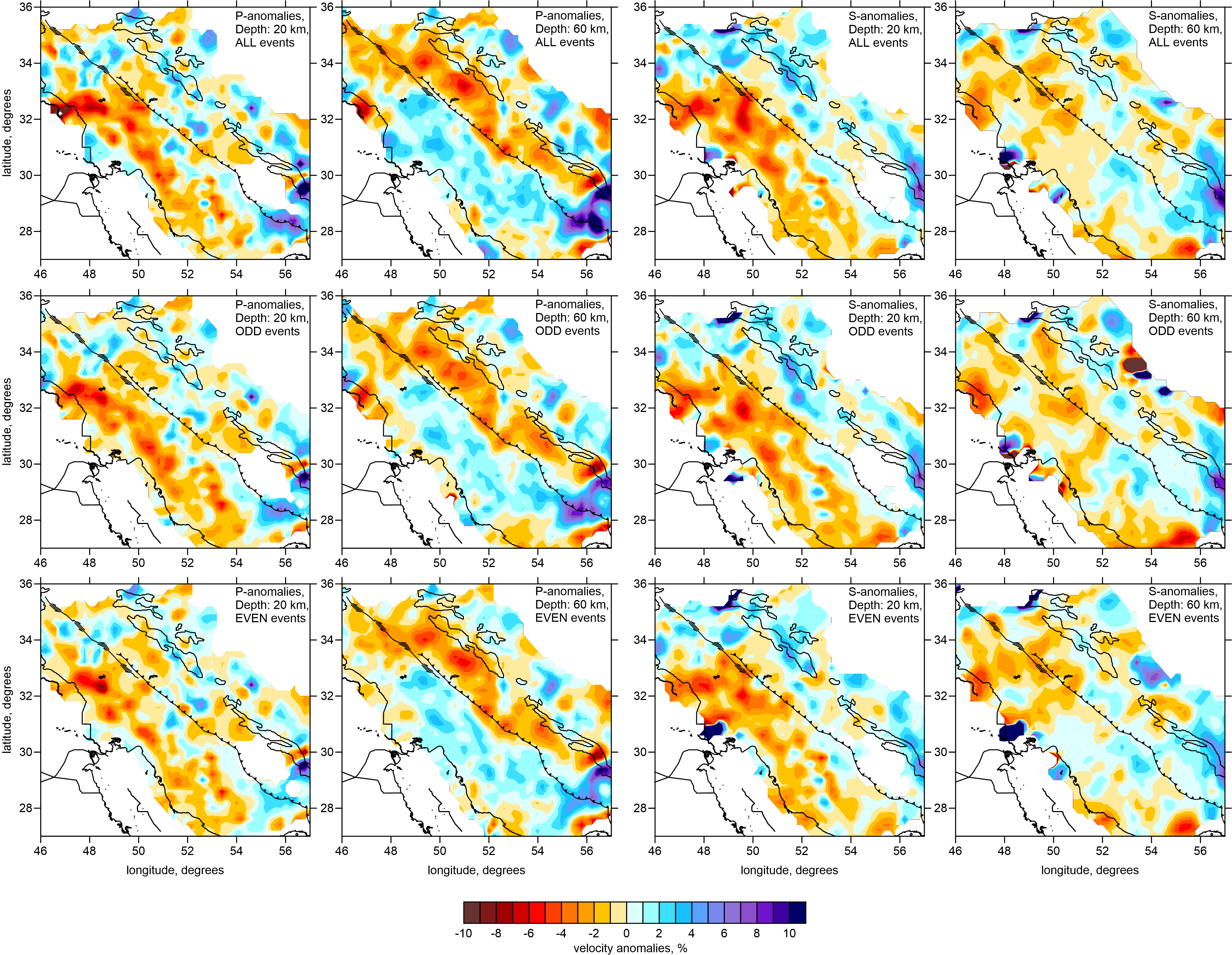


Figure S15. Results of the ‘‘Odd/Even” test for P- and S-anomalies at 20 and 60 km depth to check the contribution of random noise associated to the real data to the final tomography models. The figure was generated using the software Surfer (version 13, <http://www.goldensoftware.com/products/surfer>).


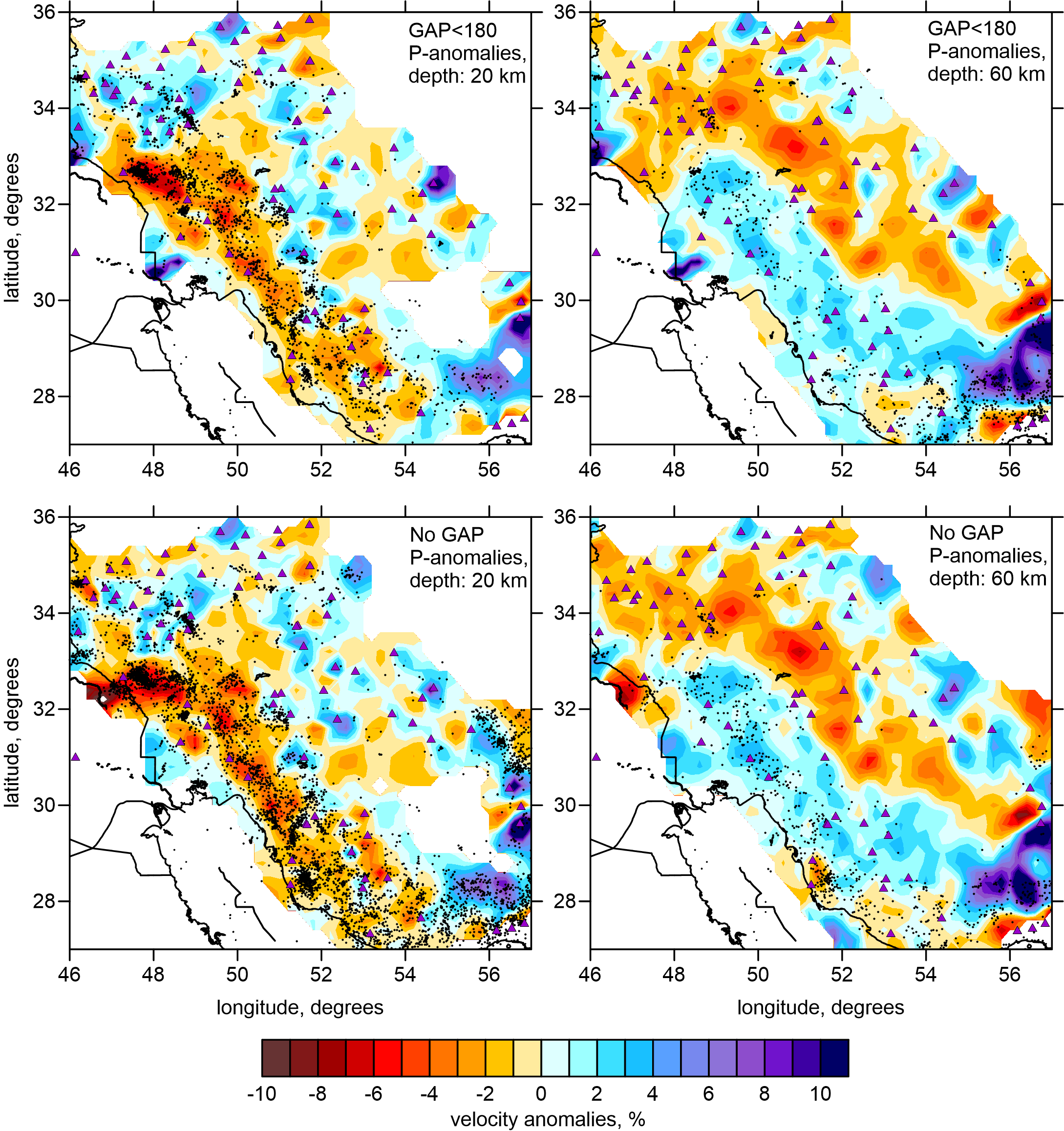


Figure S16. Comparison of the results based on different data selection criteria. Upper row: only events with azimuthal gaps of less than 180 degrees are used; lower row: no gap criterion is used. In both cases, Vp anomalies at 20 km and 60 km depth are presented. Triangles depict seismic stations and black dots are the events at the corresponding depth intervals. The figure was generated using the software Surfer (version 13, <http://www.goldensoftware.com/products/surfer>).


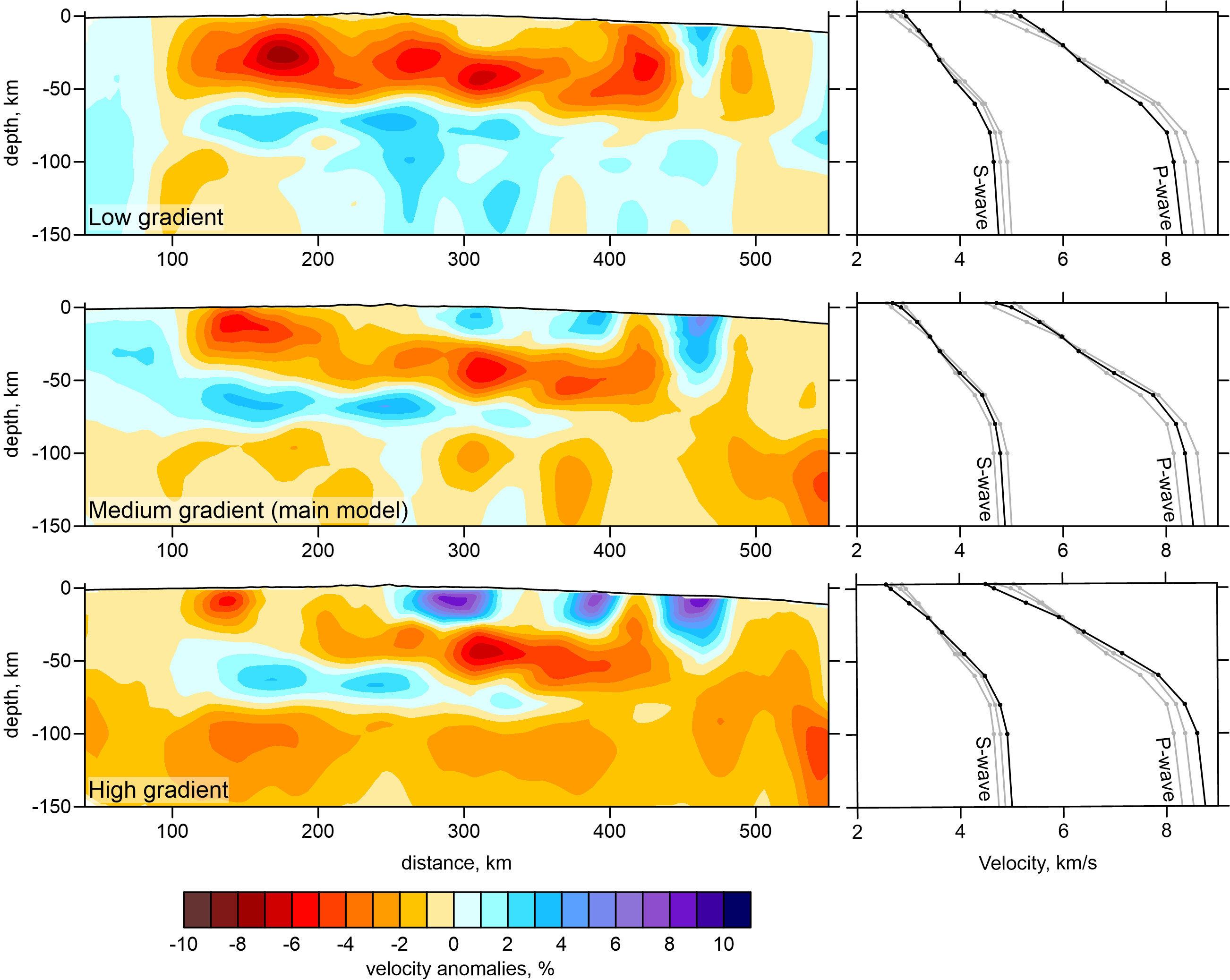


Figure S17. Results of tomographic inversion using three different reference models indicated in the right panels with black lines. In all cases, the anomalies of the P-wave velocities in Section 2 are presented (same as shown in the interpretation cartoon in Figure 3 of the main paper).

**Table S1.** Three 1-D velocity model defined by different researchers in Zagros region.

| Hatzfeld et al^22^ (Ref_01) | | Nissen et al^25^ (Ref_02) | | Yaminifard et al^26^ (Ref_03) | | Motaghi et al^27^ | |
| --- | --- | --- | --- | --- | --- | --- | --- |
| Depth, km | P velocity, km/s | Depth,  km | P velocity, km/s | Depth,  km | P velocity, km/s | Depth,  km | S velocity, km/s |
| **-3** | **5.17** | **8** | **5.3** | **0** | **5.6** | **0** | **2.5** |
| **0** | **4.66** | **14** | **5.9** | **10** | **5.8** | **20** | **3** |
| **11** | **5.84** | **20** | **6.2** | **18** | **5.9** | **30** | **3.3** |
| **19** | **6.13** | **45** | **6.5** | **22** | **6.7** | **45** | **4** |
| **46** | **8.2** |  |  |  |  | **60** | **4.4** |

**Table S2.** Average deviations of residuals and numbers of data after source locations in the starting 1D models shown in Table S1.

| 1-D velocity models | Ref_01 | Ref_02 | Ref_03 |
| --- | --- | --- | --- |
| P-residuals, s | **0.37** | **0.35** | **0.32** |
| S-residuals, s | **0.58** | **0.54** | **0.52** |
| Number of sources | **7678** | **7720** | **7783** |
| Number of P-rays | **118012** | **120551** | **123575** |
| Number of S-rays | **9261** | **10031** | **11520** |

**Table S3.** A 1-D optimized velocity model estimated for the entire Zagros collision zone.

| Ref_Zagros | | |
| --- | --- | --- |
| Depth, km | P velocity (km/s) | S velocity (km/s) |
| **-3** | **4.70** | **2.68** |
| **0** | **4.99** | **2.85** |
| **10** | **5.54** | **3.16** |
| **20** | **5.96** | **3.40** |
| **30** | **6.30** | **3.60** |
| **45** | **6.98** | **3.99** |
| **60** | **7.74** | **4.42** |
| **80** | **8.18** | **4.67** |
| **100** | **8.36** | **4.78** |
| **300** | **9.00** | **5.14** |

**Table S4.** Values of the main controlling parameters used for inversion tomography.

| Parameter | Value | |
| --- | --- | --- |
| LSQR iterations | **40** | |
| Weights for the P and S models | **1** | **1** |
| Horizontal smoothing (P, S) | **3.5** | **5.5** |
| Vertical smoothing (P, S) | **0** | **0** |
| Amplitude damping (P, S) | **0.0** | **0.0** |
| Station corrections (P and S) | **0.0001** | **0.0001** |
| Source correction: horizontal shift | **5** | |
| Source correction: vertical shift | **5** | |
| Source correction: origin time | **5** | |

**Table S5.** Values of average residuals (in the L1 norm) and variance reduction in respect to the stage of source locations in the starting 1D model.

| Iteration | P-waves | | S-waves | |
| --- | --- | --- | --- | --- |
|  | average dt, s | reduction, % | average dt, s | reduction, % |
| 1 | 0.4111 | 0 | 0.7215 | 0 |
| 2 | 0.3459 | 15.85 | 0.5985 | 17.04 |
| 3 | 0.3330 | 19.00 | 0.5564 | 22.88 |
| 4 | 0.3274 | 20.36 | 0.5303 | 26.49 |
| 5 | 0.3240 | 21.17 | 0.5164 | 28.42 |
